# Supplementary material for: Extracellular nicotinate phosphoribosyltransferase binds Toll like receptor 4 and mediates inflammation
Source: Nat Commun. 2019 Sep 11;10:4116. doi: 10.1038/s41467-019-12055-2 (PMC6739309; doi:10.1038/s41467-019-12055-2)
Supplement: Supplementary file 1 — Supplementary Information [file 41467_2019_12055_MOESM1_ESM.pdf]

Title: Extracellular nicotinate phosphoribosyltransferase binds Toll like receptor 4 and mediates inflammation

Authors: Antonella Managò[1,2,11], Valentina Audrito[1,2,11], Francesca Mazzola[3], Leonardo Sorci[4] Federica Gaudino[1,2], Katiuscia Gizzi[2], Nicoletta Vitale[5], Danny Incarnato[2], Gabriele Minazzato[6], Alice Ianniello[7], Antonio Varriale[8], Sabato D'Auria[8], Giulio Mengozzi[7], Gianfranco Politano[9], Salvatore Oliviero[2,10], Nadia Raffaelli[6,12], Silvia Deaglio[1,2,12*].

Affiliations:

[1]Department of Medical Sciences; [2]Italian Institute for Genomic Medicine, Turin, Italy; [3]Department of Clinical Sciences and [4]Department of Materials, Environmental Sciences and Urban Planning, Division of Bioinformatics and Biochemistry and [6]Department of Agricultural, Food and Environmental Sciences, Polytechnic University of Marche, Ancona, Italy; [5]Department of Molecular Biotechnology and Health Sciences, University of Turin, Italy; [7]Department of Laboratory Medicine, Azienda Ospedaliero-Universitaria Città della Salute e della Scienza, Turin, Italy; [8]Institute of Food Science, CNR, Avellino, Italy; [9]Department of Control and Computer Engineering, Politecnico di Torino, Turin, Italy; [10]Department of Life Sciences and Systems Biology, University of Turin, Italy;

[11]AM and VA contributed equally to this study

[12]NR and SD share senior authorship

***Correspondence to**: Silvia Deaglio, MD, PhD, Department of Medical Sciences, University of Turin School of Medicine & Italian Institute for Genomic Medicine (IIGM), via Nizza, 52, 10126 Torino, Italy. Email: silvia.deaglio@unito.it or silvia.deaglio@iigm.it

Supplemental Figures and legends

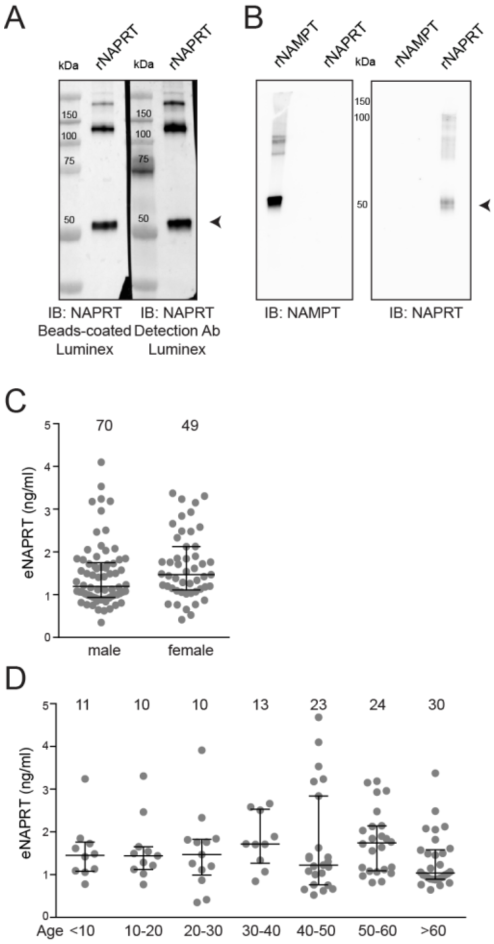

E

Tritation curve (pg/ml)		Concentration values: *Hs*NAPRT MyBioSource	Concentration values: PncB (bacterial NAPRT)
160000,0	**STD 1**	156879,63	OOR <
40000,0	**STD 2**	39999,81	OOR <
10000,0	**STD 3**	10075,66	OOR <
2500,0	**STD 4**	2430,46	OOR <
625,0	**STD 5**	664,85	OOR <
156,3	**STD 6**	141,9	OOR <
39,1	**STD 7**	42,43	OOR <
/	**Blank**	OOR <	OOR <
OOR = Out of Range			

Supplementary Figure 1

Supplementary Figure 1. eNAPRT is present in normal human plasma. (A) Western blot showing the reactivity of the NAPRT-specific antibodies used for the luminex assay when probed with rNAPRT. **(B)** Western blot showing the absence of cross-reactivity between NAMPT-specific

antibody and rNAPRT and NAPRT-specific antibody and rNAMPT. **(C-D)** eNAPRT concentrations (ng/ml) measured by luminex in a validation cohort of plasma from HD divided according to sex **(C)** and age **(D)**. **(E)** Table showing the specificity of luminex assay to recognize the recombinant *Hs*NAPRT and not the bacterial one (PncB).

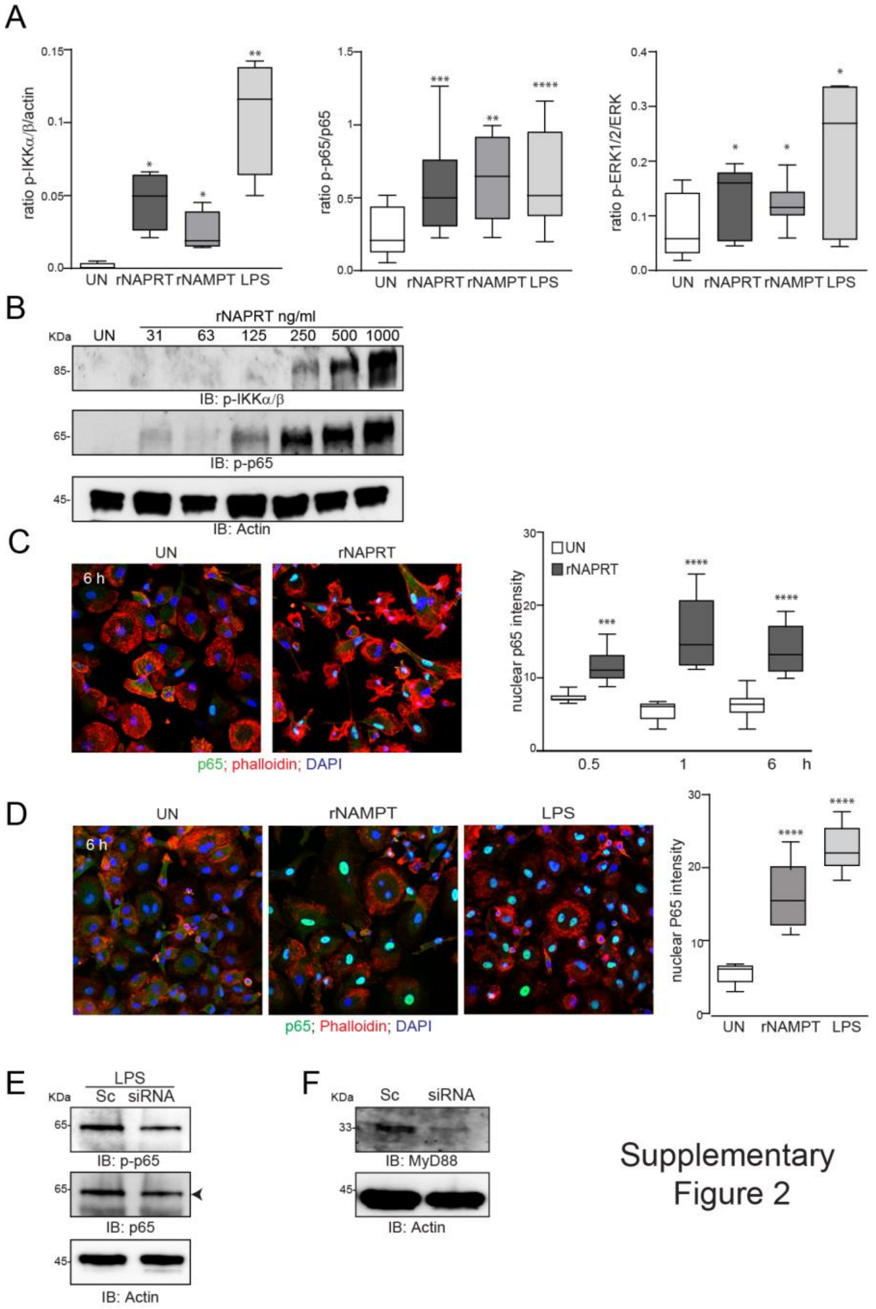

Supplementary Figure 2. NF-Kb signaling activation. (A) Box plots represent band quantification using ImageQuant software of p-IKKβ/α/Actin (n=4), p-p65/p65 (n=12 for rNAPRT and n=9 for rNAMPT), pERK1/2/ERK (n=6), paired t test. **(B)** Western blot showing dose-dependent NF-KB activation (p-IKKα/β and p-p65) upon rNAPRT macrophage exposure for 30 minutes at 37°C. **(C)** Confocal microscopy analysis of p65 staining (green fluorescence) in human macrophages treated with rNAPRT (1 µg/ml, 37°C, 6 hours) [Original magnification was 63x]. On the right side, relative

quantification of nuclear p65 intensity of macrophages from at least 3 different HD (at least 3 different fields were counted) treated with rNAPRT (1 µg/ml, 30 minutes 37°C, 1 hour or 6 hours). Mann-Whitney test. **(D)** p65 localization in human macrophages upon treatment (1 hour, 37°C) with rNAMPT (1 µg/ml) and LPS (2 µg/ml) evaluated by confocal microscopy (original magnification 63x). Graph represents the relative quantification of nuclear p65 intensity of 3 different HD (at least 3 different fields were counted). In both **(C)** and **(D)**, cells were counterstained with Alexa568-conjugated phalloidin and with DAPI to highlight the cytoskeleton and the nucleus, respectively. All samples were analyzed using a TCS SP5 laser scanning confocal microscope (Leica Microsystems). Images were acquired with LAS AF Version Lite 2.4 software and processed with Photoshop (Adobe Systems). Pixel intensities were calculated with ImageJ software (freely downloadable at http://rsbweb.nih.gov/ij/). Results are reported as box plots, where the top and bottom margins of the box define the 25th and 75th percentile, the line in the box defines the median and the error bars define the minimum and maximum of all data.

(E) Western blot analysis of p-p65 in scramble (sc) or MyD88 siRNA-silenced macrophages upon treatment (30 minutes, 37°C) with LPS (2 µg/ml, n=7). **(F)** Western blot analysis of MyD88 in scramble (sc) or MyD88 siRNA-silenced macrophages in basal conditions. Paired t test. Results are reported as box plots, where the line in the box defines the median and the error bars define the minimum and maximum of all data. Source data are provided as a Source Data file.

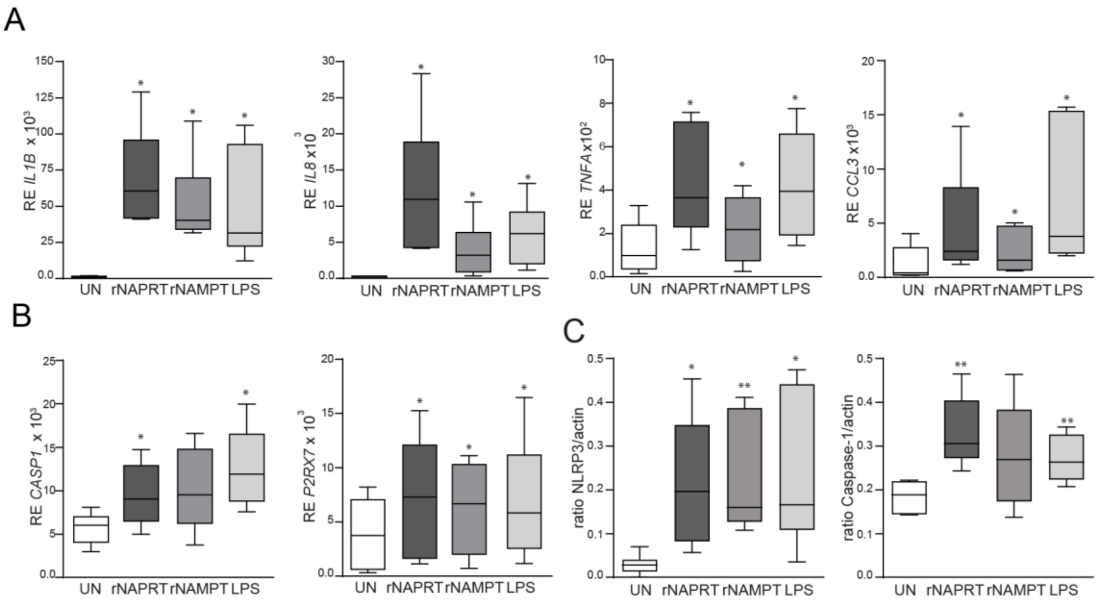

Supplementary
Figure 3

Supplementary Figure 3. NF-kB pathway activation by eNAPRT is followed by the induction of transcription and secretion of pro-inflammatory cytokines. (A) Box plots showing mRNA expression levels of *IL1B, IL8, TNFA* and *CCL3* evaluated by qRT-PCR in RNA from HD macrophages (n=6) treated with rNAPRT (1 µg/ml), rNAMPT (1 µg/ml) and LPS (2 µg/ml, left panels) for 15 hours at 37°C. **(B)** Box plots showing mRNA expression levels of *CASP1,* and *P2RX7* evaluated by qRT-PCR in the macrophage RNA preparations. Paired t test. **(C)** Box plot represents band quantification of NLRP3 and Caspase-1 in at least n=5 HD macrophages upon treatment (6 hours, 37°C) with rNAPRT (1 µg/ml), rNAMPT (1 µg/ml) or LPS (2 µg/ml) . Paired t test. Results are reported as box plots, where the line in the box defines the median and the error bars define the minimum and maximum of all data. Source data are provided as a Source Data file.

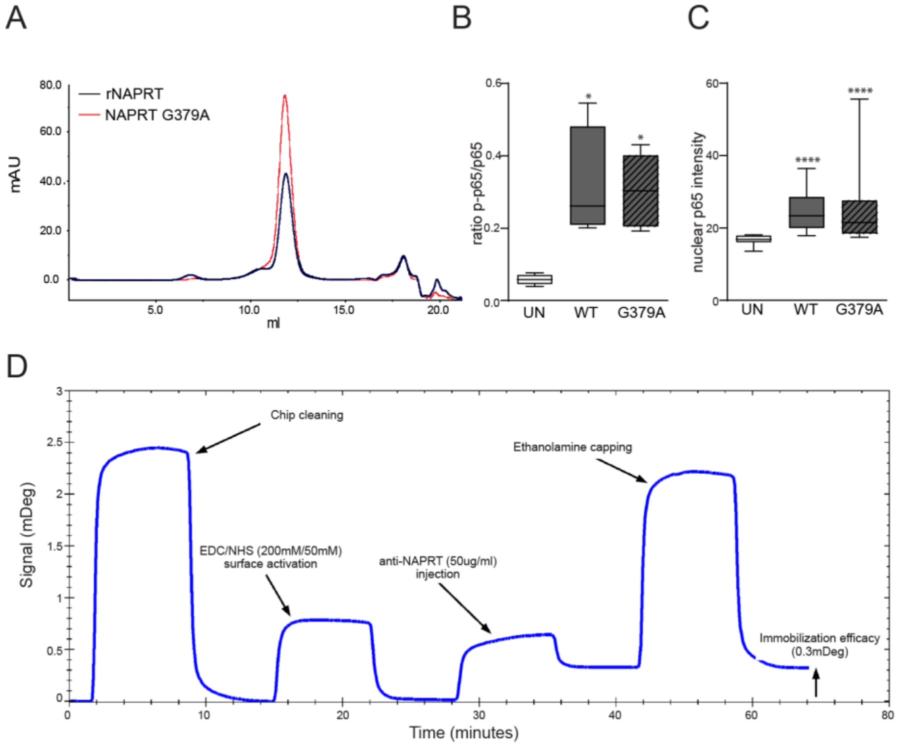

Supplementary Figure 4

Supplementary Figure 4. eNAPRT binds to TLR4. (A) Gel filtration chromatography of rNAPRT and G379A mutant. Elution profiles of the proteins at 280 nm are shown. **(B)** Box plot represents western blot quantification obtained by ImageQuant of p-p65/p65 p65 in HDs macrophages (n=5) upon treatment (30 minutes, 37°C) with rNAPRT or the G379A mutant. All recombinant proteins were used at the concentration of 1 µg/ml. Wilcoxon test. **(C)** Box plot shows relative quantification of nuclear p65 mean fluorescence intensity (n=3) in macrophages treated as above. Pixel intensities were calculated with ImageJ software (freely downloadable at http://rsbweb.nih.gov/ij/). Mann-Whitney test. **(D)** SPR immobilization profile of anti-NAPRT1 on the SPR102-CMD-2D chip. Results are reported as box plots, where the line in the box defines the median and the error bars define the minimum and maximum of all data. Source data are provided as a Source Data file.

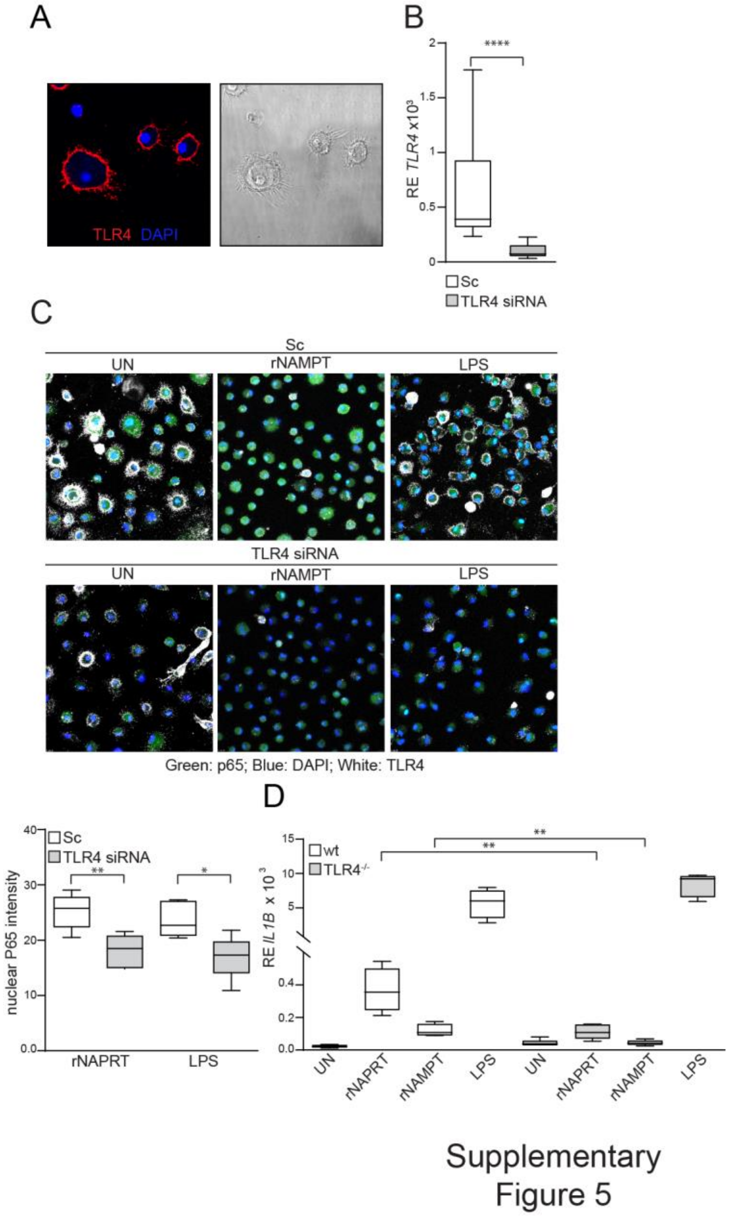

Supplementary Figure 5. rNAPRT induces "inflammosome" pathways via TLR4 binding in macrophages. (A) Confocal microscopy analysis showing that PBMC-derived human macrophages express TLR4 on the plasma membrane. Cells were counterstained with DAPI to highlight the nucleus. Original magnification 63x. Images were acquired with LAS AF Version Lite 2.4 software and processed with Photoshop (Adobe Systems). **(B)** Box plots reporting *TLR4* mRNA expression

levels in human macrophages 72 hours after transfection with a scramble control siRNA (Sc) or a TLR4 siRNA, analyzed by qRT-PCR. Paired t test. **(C)** Confocal images showing p65 localization (green fluorescence) in macrophages transfected with a scramble control siRNA (sc) or a TLR4 siRNA (white fluorescence for TLR4 staining). Treatment (30 minutes, 37°C) with rNAMPT (1 µg/ml) and LPS (2 µg/ml). Left bottom panels showed box plots of nuclear p65 intensity (n=3). Mann-Whitney test. Cells were counterstained with DAPI. Original magnification 63x. Images were acquired with LAS AF Version Lite 2.4 software and processed with Photoshop (Adobe Systems). Pixel intensities were calculated with ImageJ software (freely downloadable at http://rsbweb.nih.gov/ij/). **(D)** Box plots showing mRNA expression levels of *IL1B* evaluated by qRT-PCR in RNA from macrophages derived from TLR4-/- (n=6) or wt (n=4) mice treated with rNAPRT (1 µg/ml), rNAMPT (1 µg/ml) and LPS (2 µg/ml) for 15 hours at 37°C, unpaired t test. Results are reported as box plots, where the line in the box defines the median and the error bars define the minimum and maximum of all data. Source data are provided as a Source Data file.

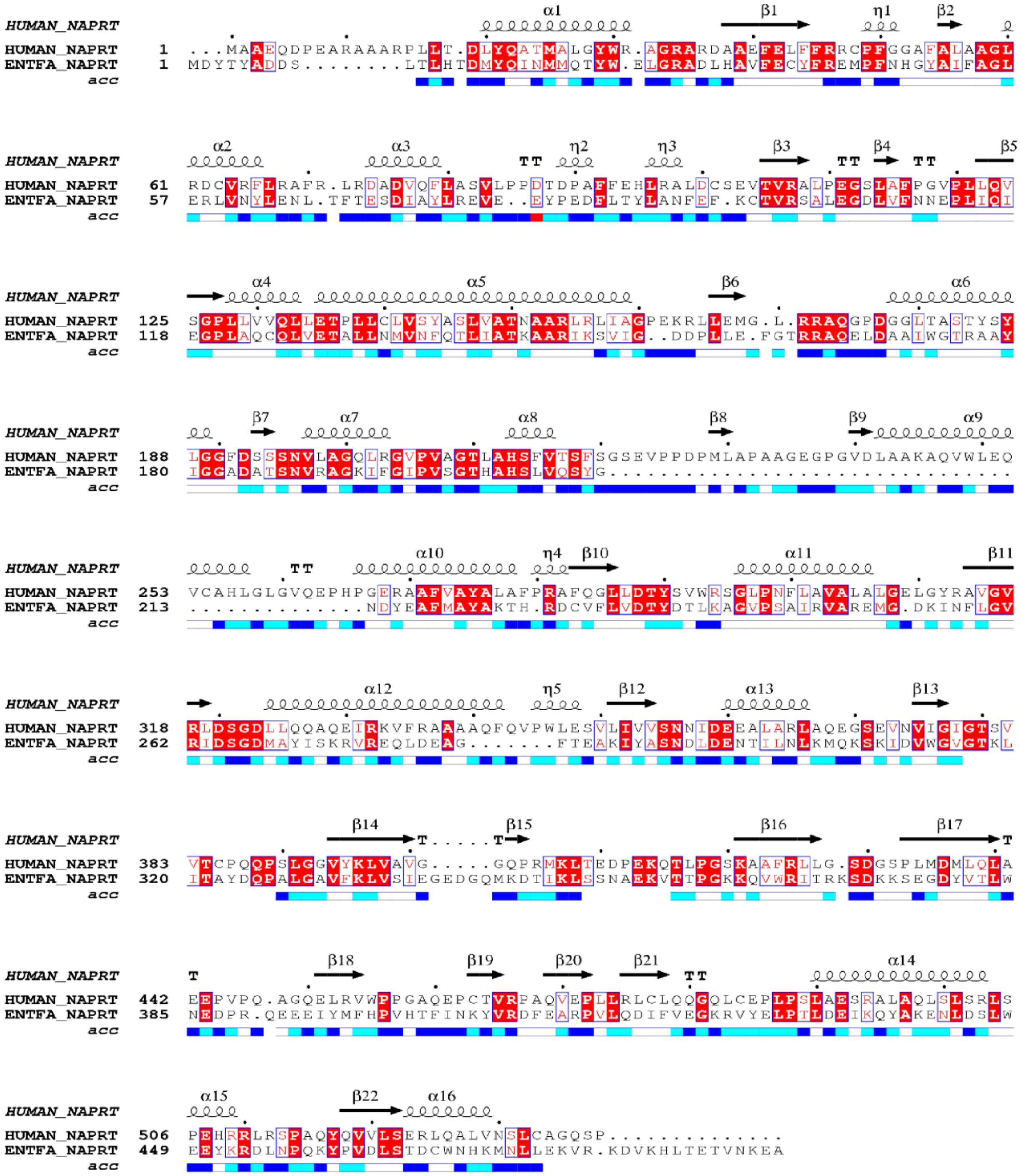

Supplementary Figure 6

Supplementary Figure 6. Structural comparison of bacterial and human NAPRT. Structure-based sequence alignment of human and *E. faecalis* NAPRT. Secondary structure elements of the human NAPRT are represented on the top of alignment by spirals (α and 310 helices) and arrows (β strands) and turns by TT. The 310 helices are labeled η. Similar amino acids are boxed, and identity is indicated by a solid red background color. Solvent accessibility is rendered by a bar below the sequence (blue is accessible, cyan is intermediate, white is buried).

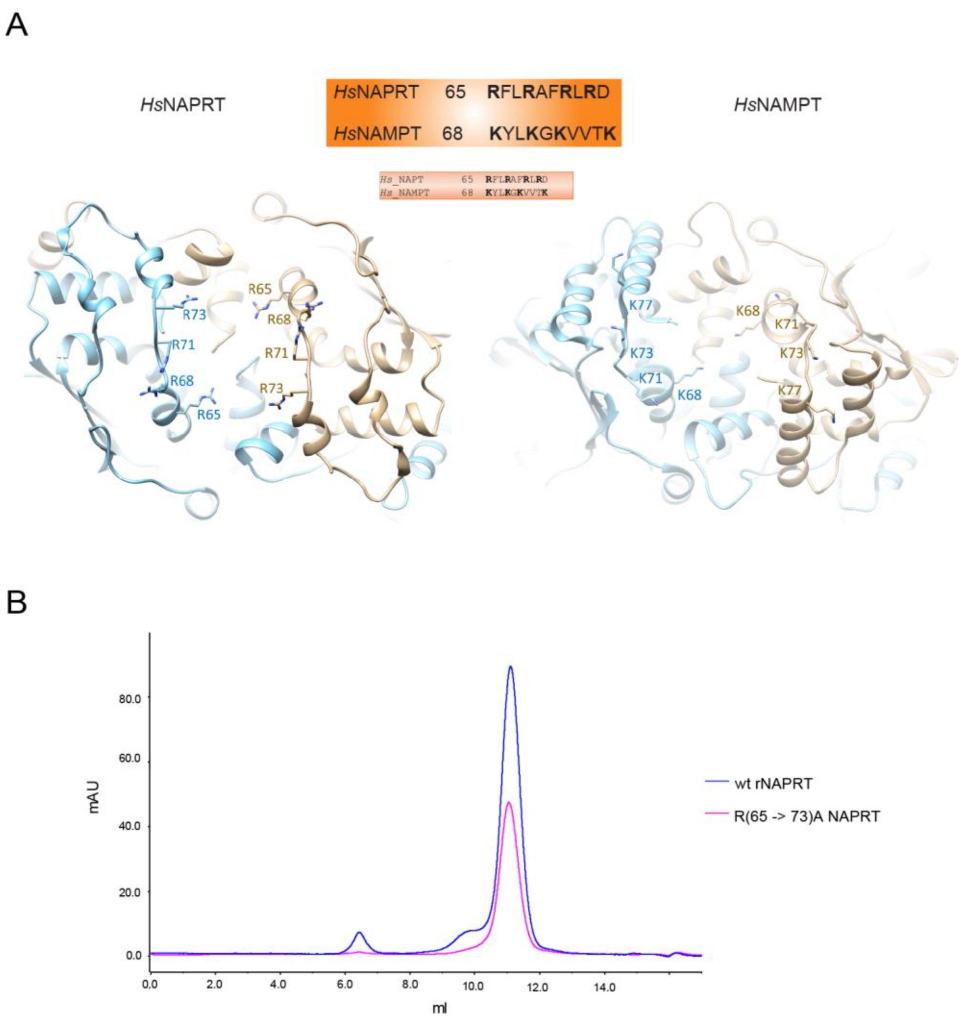

Supplementary Figure 7

Supplementary Figure 7. Structural determinants of rNAPRT and rNAMPT involved in TLR4 binding. (A) Close-up view of the homologous mouth-like, positively-charged areas in human NAPRT and NAMPT. These surface-exposed corresponding regions were obtained from structural superimposition. (B) Elution profiles at 280 nm from gel filtration chromatography of wild type rNAPRT and the R(65->73)A NAPRT mutant.

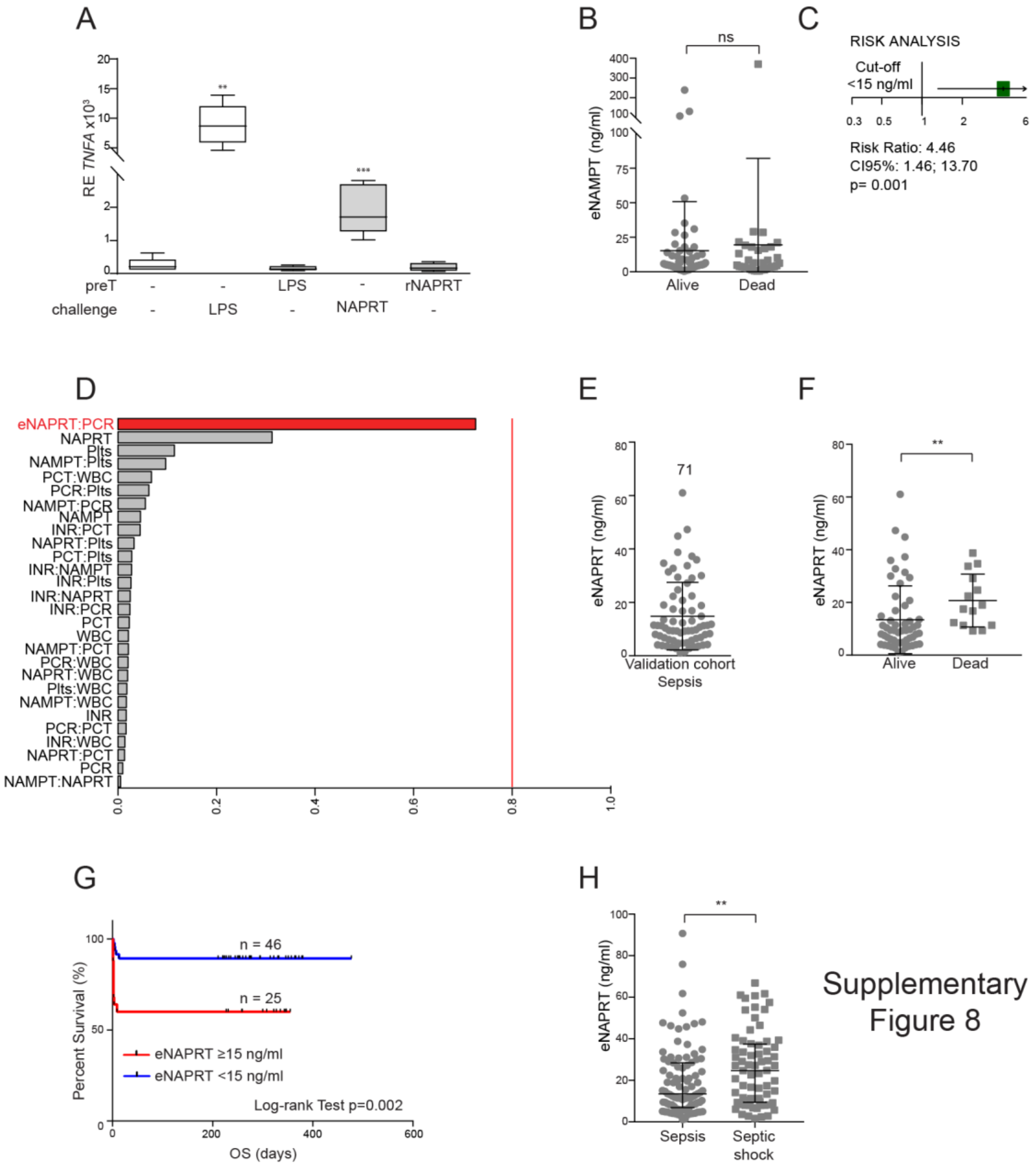

Supplementary Figure 8. **eNAPRT in septic patients. (A)** Box plots showing mRNA expression levels of *TNFA* evaluated by qRT-PCR in RNA from HD macrophages (at least n=4) treated as follow: 30 hours of pre-conditioning (preT) with LPS 10 ng/ml or rNAPRT 10 ng/ml followed by 6 hours of challenging with LPS 1 µg /ml or rNAPRT 1 µg /ml. Paired t test. **(B)** Scatter dot-plots showing eNAMPT levels in septic patients who survived and those who did not. Mann-Whitney test. **(C)** Analysis showing the risk ratio of mortality applying the eNAPRT cut-off of 15ng/ml. **(D)** GLM analysis to test most informative associations of eNAPRT between biochemical markers in septic

patients. Abbreviations: C-reactive protein (CRP), procalcitonin (PCT), white blood cells count (WBC), platelets (Plts), International Normalized Ratio (INR) derived from prothrombin time (PT). **(E)** Scatter dot plots showing eNAPRT levels measured by luminex on plasma/sera samples from a validation cohort of 71 septic patients. **(F)** Scatter dot plot showing eNAPRT levels according to outcome in the septic validation cohort, Mann-Whitney test. **(G)** Kaplan-Meyer curves showing overall survival (OS) of the cohort of 71 septic patients divided on the basis of eNAPRT levels. Log-rank test shows statistical significance. **(H)** Scatter dot plot showing eNAPRT levels in patients with sepsis or septic shock, Mann-Whitney test. Results are reported as box plots, where the line in the box defines the median and the error bars define the minimum and maximum of all data. Source data are provided as a Source Data file.

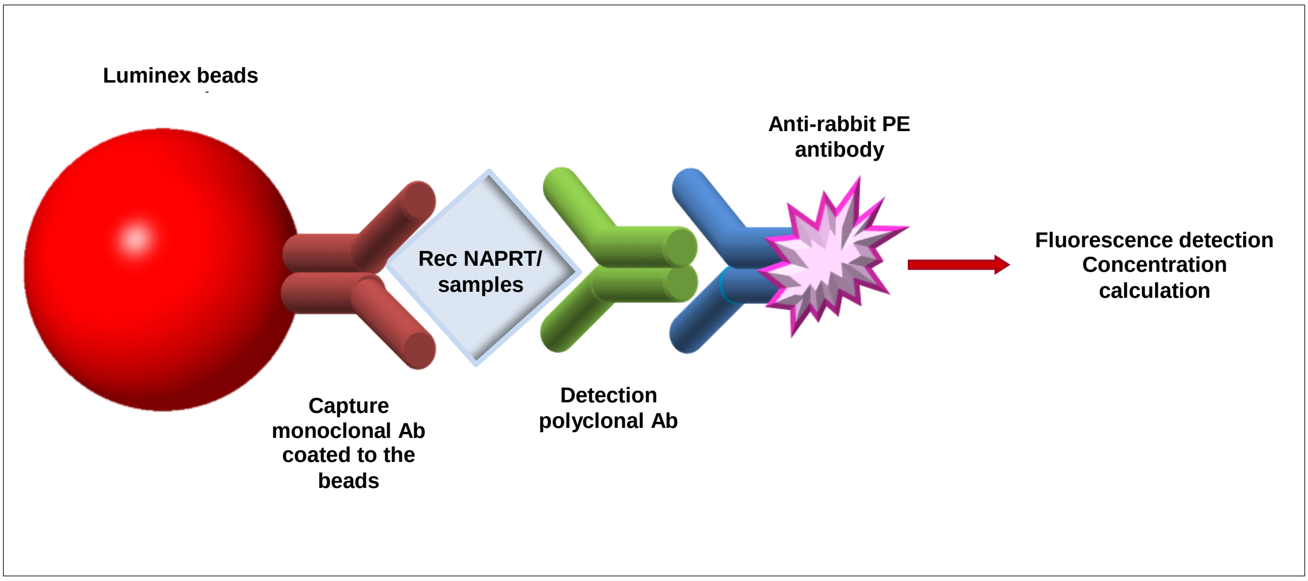

NAPRT luminex assay workflow

Supplementary Figure 9. **Schematic representation of NAPRT luminex assay.** A monoclonal antibody anti-NAPRT (ProteinTech), chemically coated to luminex beads was used for bind soluble NAPRT present in plasma/sera or supernatants samples. A polyclonal antibody anti-NAPRT (MyBioSource) and the secondary anti-rabbit PE were then used to obtain the direct quantification of the fluorescence intensity that correlates with the amount of NAPRT in the sample.

Commercially available rNAPRT-GST-tag (MyBioSource MBS969577) was used to build a titration curve. The detection range of the assay is from 10 pg/ml to 500 ng/ml.

Supplemental Tables

Supplementary Table 1

Coupling Luminex NAPRT1		Fluorescence	Concentration
Tritation curve (pg/ml) rNAMPT			NAMPT
1360500,0	**STD 1**	231,5	OOR <
340121,0	**STD 2**	227	OOR <
85030,3	**STD 3**	231,5	OOR <
21257,6	**STD 4**	226	OOR <
5314,4	**STD 5**	292,5	OOR <
1328,6	**STD 6**	272,5	OOR <
332,2	**STD 7**	327	OOR <
83,0	**STD 8**	264,0	OOR <

Coupling Luminex NAMPT		Fluorescence	Concentration
Tritation curve (pg/ml) rNAMPT			NAMPT
1360500,0	**STD 1**	25659,5	1055000,0
340121,0	**STD 2**	24404,0	294337,0
85030,3	**STD 3**	21011,5	97495,3
21257,6	**STD 4**	10606,0	20245,6
5314,4	**STD 5**	3495,8	5485,0
1328,6	**STD 6**	876,5	1307,1
332,2	**STD 7**	341,3	331,8
83,0	**STD 8**	235,0	85,0

OOR = Out of Range

Specificity of NAPRT Luminex assay. Luminex assay for NAPRT (beads coated with anti-NAPRT antibody) did not recognize rNAMPT, while, as a control, commercial luminex for NAMPT (beads coated with anti-NAMPT antibody) detected the rNAMPT used for the titration curve.
