# Supplementary material for: Extracellular nicotinate phosphoribosyltransferase binds Toll like receptor 4 and mediates inflammation
Source: Nat Commun. 2019 Sep 11;10:4116. doi: 10.1038/s41467-019-12055-2 (PMC6739309; doi:10.1038/s41467-019-12055-2)
Supplement: Supplementary file 3 — Reporting Summary [file 41467_2019_12055_MOESM3_ESM.pdf]

nature research

Corresponding author(s): Silvia Deaglio

Last updated by author(s): 2019-7-17

Reporting Summary

Nature Research wishes to improve the reproducibility of the work that we publish. This form provides structure for consistency and transparency in reporting. For further information on Nature Research policies, see Authors & Referees and the Editorial Policy Checklist.

Statistics

For all statistical analyses, confirm that the following items are present in the figure legend, table legend, main text, or Methods section.

n/a	Confirmed	
☐	☒	The exact sample size (*n*) for each experimental group/condition, given as a discrete number and unit of measurement
☐	☒	A statement on whether measurements were taken from distinct samples or whether the same sample was measured repeatedly
☐	☒	The statistical test(s) used AND whether they are one- or two-sided<br>*Only common tests should be described solely by name; describe more complex techniques in the Methods section.*
☒	☐	A description of all covariates tested
☒	☐	A description of any assumptions or corrections, such as tests of normality and adjustment for multiple comparisons
☐	☒	A full description of the statistical parameters including central tendency (e.g. means) or other basic estimates (e.g. regression coefficient) AND variation (e.g. standard deviation) or associated estimates of uncertainty (e.g. confidence intervals)
☐	☒	For null hypothesis testing, the test statistic (e.g. F, t, r) with confidence intervals, effect sizes, degrees of freedom and P value noted<br>*Give P values as exact values whenever suitable.*
☒	☐	For Bayesian analysis, information on the choice of priors and Markov chain Monte Carlo settings
☒	☐	For hierarchical and complex designs, identification of the appropriate level for tests and full reporting of outcomes
☐	☒	Estimates of effect sizes (e.g. Cohen's *d*, Pearson's *r*), indicating how they were calculated

Our web collection on statistics for biologists contains articles on many of the points above.

Software and code

Policy information about availability of computer code

Data collection	Data were collected using Windows Office Excel 2010
Data analysis	tatistical analysis were performed using GraphPad version 7.0 (GraphPad Software Inc) and R (R Core Team (https://www.r-project.org/). The 3D structures of the proteins were aligned using PROMALS3D538 and the figures were produced using ESPript639.

For manuscripts utilizing custom algorithms or software that are central to the research but not yet described in published literature, software must be made available to editors/reviewers. We strongly encourage code deposition in a community repository (e.g. GitHub). See the Nature Research guidelines for submitting code & software for further information.

Data

Policy information about availability of data

All manuscripts must include a data availability statement. This statement should provide the following information, where applicable:

- Accession codes, unique identifiers, or web links for publicly available datasets
- A list of figures that have associated raw data
- A description of any restrictions on data availability

All data are available within the manuscript and in the supplemental materials and raw data are available in data source file

Field-specific reporting

Please select the one below that is the best fit for your research. If you are not sure, read the appropriate sections before making your selection.

☒ Life sciences ☐ Behavioural & social sciences ☐ Ecological, evolutionary & environmental sciences

Life sciences study design

All studies must disclose on these points even when the disclosure is negative.

Sample size	Sample size corresponds to biological replicates needed to obtain statistical significance. At least four different independent samples per condition were analyzed (except for RNA-seq and P65 immunofluorescence of TLR4 silenced macrophages, that were performed on 3 different samples).
Data exclusions	No data were excluded from the analyses and raw data are available upon request to the corresponding author.
Replication	Each experiment was replicated at least four times (independent biological replicates) to verify the hypothesis (except for RNA-seq and P65 immunofluorescence of TLR4 silenced macrophages, that were performed on 3 different samples).
Randomization	NA
Blinding	NA

Reporting for specific materials, systems and methods

We require information from authors about some types of materials, experimental systems and methods used in many studies. Here, indicate whether each material, system or method listed is relevant to your study. If you are not sure if a list item applies to your research, read the appropriate section before selecting a response.

Materials & experimental systems

n/a	Involved in the study	
☐	☒	Antibodies
☒	☐	Eukaryotic cell lines
☒	☐	Palaeontology
☐	☒	Animals and other organisms
☐	☒	Human research participants
☐	☒	Clinical data

Methods

n/a	Involved in the study	
☒	☐	ChIP-seq
☒	☐	Flow cytometry
☒	☐	MRI-based neuroimaging

Antibodies

Antibodies used	Antibodies used for western blot and immunoprecipitation<br>The following antibodies were used for western blot: anti-NAMPT (A300-779A, Bethyl Laboratories, Montgomery, TX), anti-NAPRT1 (NBP1-87243, Novus Biologicals, Littleton, CO; AMAB90725 Atlas; 66159-I-Ig ProteinTech, Manchester, UK and MBS1491066 MyBioSource), anti-phospho-p65 (Ser536, #3033S), anti-p65 (#8242S), anti-phospho-IKKα/β (Ser176/180, #2697), anti-IRAK1 (#4504), anti-NLRP3 (#13158), anti-Caspase-1 (#3866) and Cyclophilin A (#2175) all from Cell Signaling Technologies (Danvers, MA), anti-pERK1/2 (pT202/Y204, 612358) and anti-panERK (610123) both from BD Biosciences (East Rutheford, NJ), anti-MyD88 (sc-11356, Santa Cruz Biotechnology, Dallas, TX) and anti-actin horseradish peroxidase (HRP)-conjugated (ab20272, Abcam, Cambridge, UK). Secondary reagents were: goat anti-mouse IgG-HRP-conjugated (Perkin Elmer, Waltham, MA), goat anti-rabbit HRP-conjugated (Santa Cruz Biotechnology).<br>Antibodies used for confocal microscopy<br>Antibodies used for immunofluorescence were: anti-NAPRT1 (NBP1-87243, 1:100 Novus Biological) anti-p65 (sc-8008, 1:100 from Santa Cruz Biotechnology), anti-TLR4 (NB-10056566, 1:100 from Novus Biologicals), anti-CD68 Alexa Fluor-488 (333812, 5 µl for well from Biolegend, San Diego, CA), anti-CD11b (HPA002274, 1:200 from Sigma-Aldrich, Saint Louis, MO). Secondary reagents were: goat anti-mouse IgG AlexaFluor488-conjugated (1:50) and goat anti-rabbit IgG AlexaFluor488-conjugated (1:100, both from Thermo-Fisher, Waltham, MA). After the primary and secondary antibodies, cells were counter-stained with AlexaFluor 568-conjugated phalloidin (1:100) and 4',6-Diamidino-2-phenylindole (DAPI, 1:30,000, both from Thermo-Fisher).
Validation	All the antibodies used in the study are commercial and previously validated by the manufacturer. Commercial anti-NAMPT and anti-NAPRT antibodies were validated in this study for crossreactivity and for human specificity for luminex assay setting (patent I0174545).

Animals and other organisms

Policy information about studies involving animals; ARRIVE guidelines recommended for reporting animal research

Laboratory animals	TLR4-/- mice [B6(Cg)-Tlr4tm1.2Karp/J] or C57BL/6 wt were purchased from The Jackson Laboratory (Bar Harbor, ME).
Wild animals	NA

Field-collected samples	NA
Ethics oversight	Italian Ministry of Health (#118/2018-PR)

Note that full information on the approval of the study protocol must also be provided in the manuscript.

Human research participants

Policy information about studies involving human research participants

Population characteristics	Normal donors: both genders; age range 2-74.<br>Cancer patients, including solid tumors (lung, prostate and bladder cancer, metastatic melanoma and mesothelioma) and hematological malignancies (chronic lymphocytic leukemia, myeloma and diffuse large cell lymphoma): both genders; adulthood.<br>Septic patients: both genders; adulthood.
Recruitment	Plasma from healthy donors were obtained from the local Blood Bank.<br>All patients with a diagnosis of sepsis and septic shock admitted to the emergency room of the Città della Salute e della Scienza Hospital (Turin, Italy) between October 2016 and April 2017 and followed-up until January 2018, were enrolled in the study.<br>Samples from patients with cancer were provided by clinicians, cited in the acknowledgement section.
Ethics oversight	Ethical committee of the Città della Salute e della Scienza Hospital (Turin, Italy).

Note that full information on the approval of the study protocol must also be provided in the manuscript.

Clinical data

Policy information about clinical studies

All manuscripts should comply with the ICMJE guidelines for publication of clinical research and a completed CONSORT checklist must be included with all submissions.

Clinical trial registration	NA
Study protocol	NA
Data collection	The clinical information presented in the study (LDH, creatinine, CRP, procalcitonin levels; whte blood cells and platelets count) were obtained from the clinical chart at the time of admission. Survival data were obtained by checking the hospital registry.
Outcomes	NA
