# Supplementary material for: Extracellular nicotinate phosphoribosyltransferase binds Toll like receptor 4 and mediates inflammation
Source: Nat Commun. 2019 Sep 11;10:4116. doi: 10.1038/s41467-019-12055-2 (PMC6739309; doi:10.1038/s41467-019-12055-2)
Supplement: Supplementary file 4 — Description of Additional Supplementary Files [file 41467_2019_12055_MOESM4_ESM.pdf]

Description of Additional Supplementary Files

File Name: Supplementary Data 1. RNA-seq data of differentially expressed genes.

Description: Genes list of the 555 NAPRT/NAMPT-commonly regulated genes derived from RNA-seq experiment

File Name: Supplementary Data 2. RNA-seq data of differentially expressed genes.

Description: Genes list of the 471 NAPRT exclusively regulated genes derived from RNA-seq experiment

File Name: Supplementary Data 3. RNA-seq data of differentially expressed genes.

Description: Genes list of the 71 NAMPT exclusively regulated genes derived from RNA-seq experiment
