# Supplementary material for: Extracellular nicotinate phosphoribosyltransferase binds Toll like receptor 4 and mediates inflammation
Source: Nat Commun. 2019 Sep 11;10:4116. doi: 10.1038/s41467-019-12055-2 (PMC6739309; doi:10.1038/s41467-019-12055-2)
Supplement: Supplementary file 8 — Source Data [file 41467_2019_12055_MOESM8_ESM.zip › Unprocessed images of all gels and blots.pptx]

## Slide 1
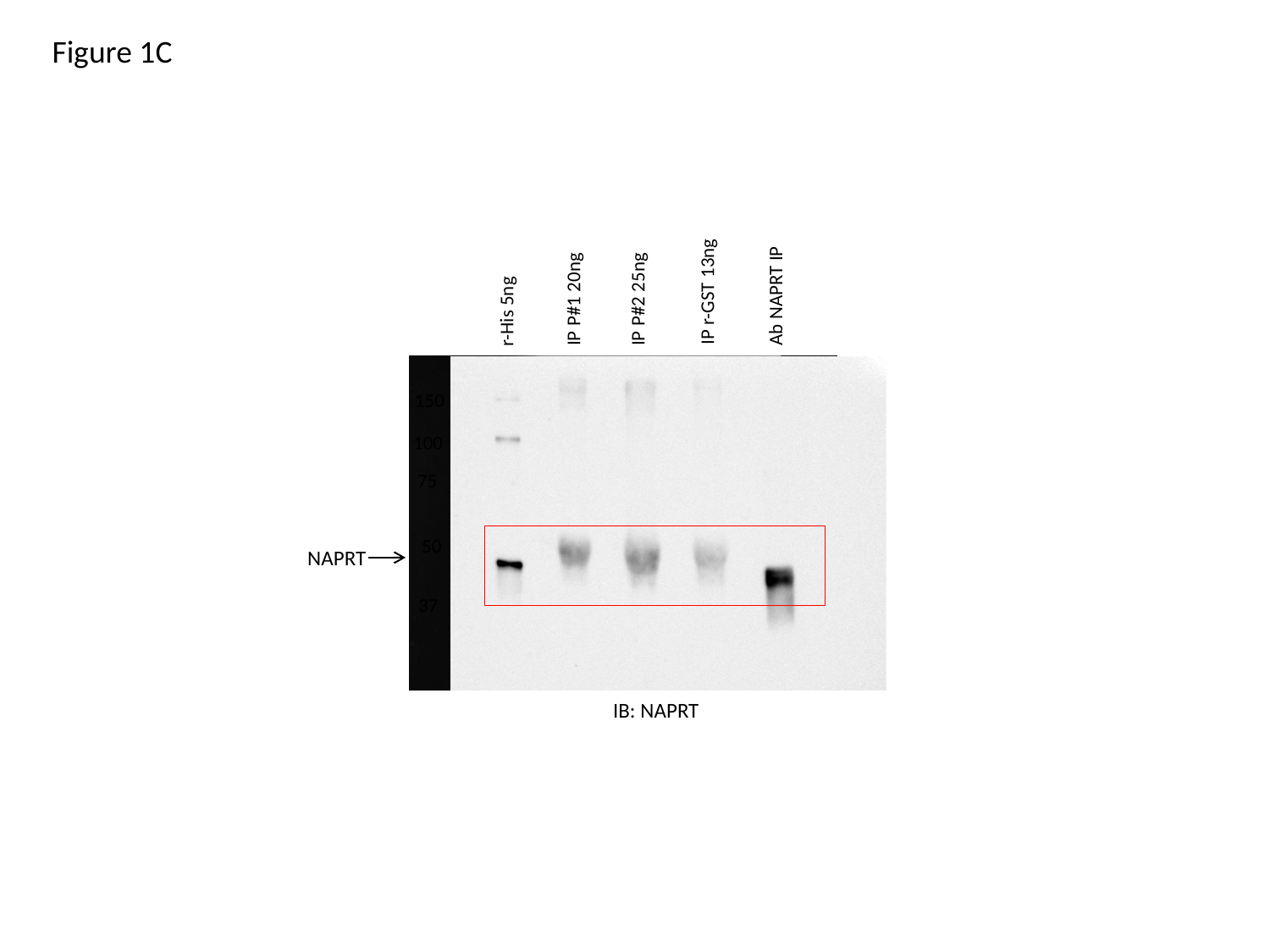

Figure 1C
IP r-GST 13ng
Ab NAPRT IP
IP P#1 20ng
IP P#2 25ng
r-His 5ng
150
100
75
50
NAPRT
37
IB: NAPRT

## Slide 2
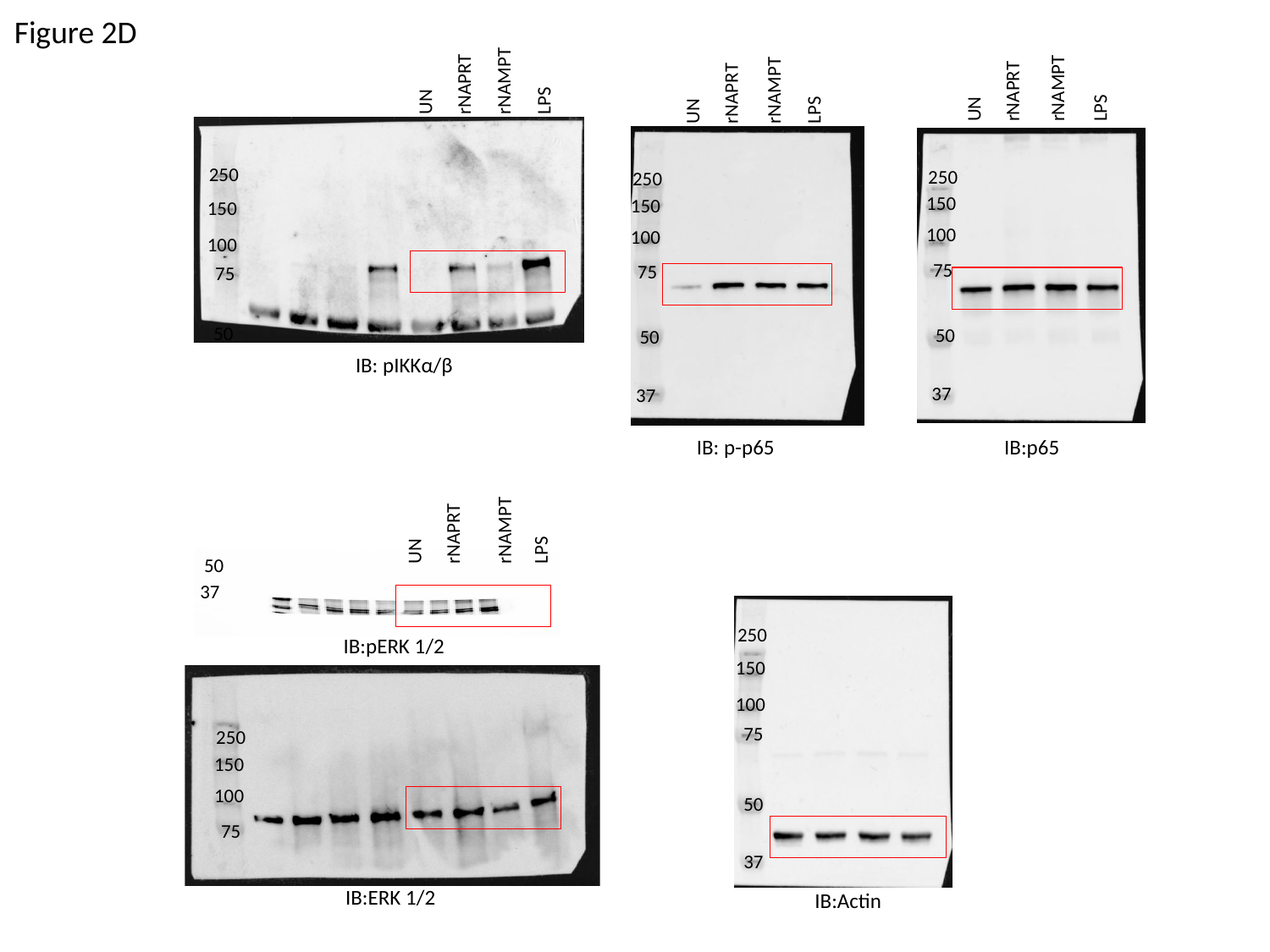

Figure 2D
rNAPRT
rNAMPT
LPS
UN
rNAPRT
rNAMPT
LPS
UN
rNAPRT
rNAMPT
LPS
UN
250
250
250
150
150
150
100
100
100
75
75
75
50
50
50
IB: pIKKα/β
37
37
IB: p-p65
IB:p65
rNAPRT
rNAMPT
LPS
UN
50
37
250
IB:pERK 1/2
150
100
75
250
150
100
50
75
37
IB:ERK 1/2
IB:Actin

## Slide 3
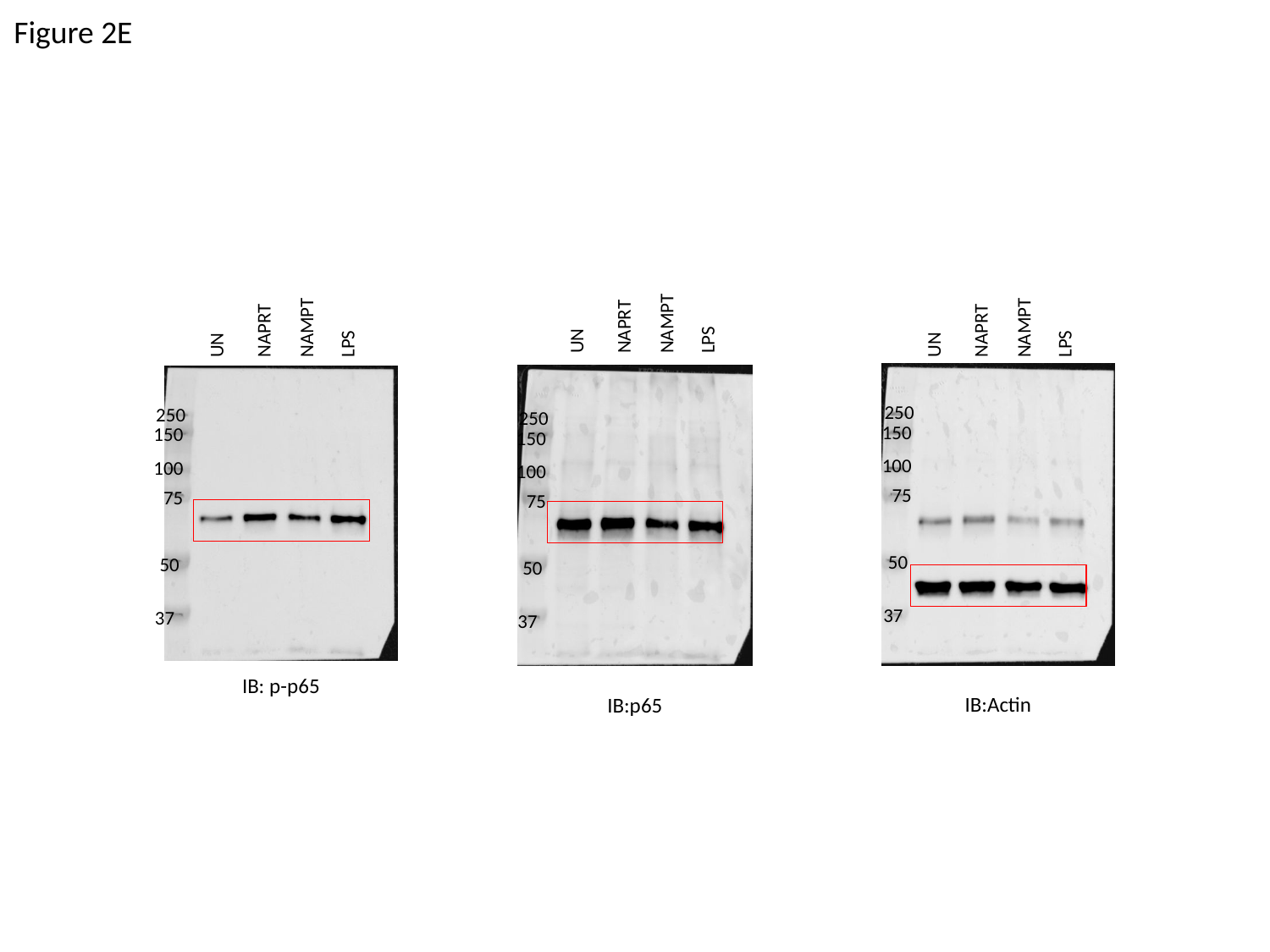

Figure 2E
NAPRT
NAMPT
LPS
UN
NAPRT
NAMPT
LPS
NAPRT
NAMPT
LPS
UN
UN
250
250
250
150
150
150
100
100
100
75
75
75
50
50
50
37
37
37
IB: p-p65
IB:Actin
IB:p65

## Slide 4
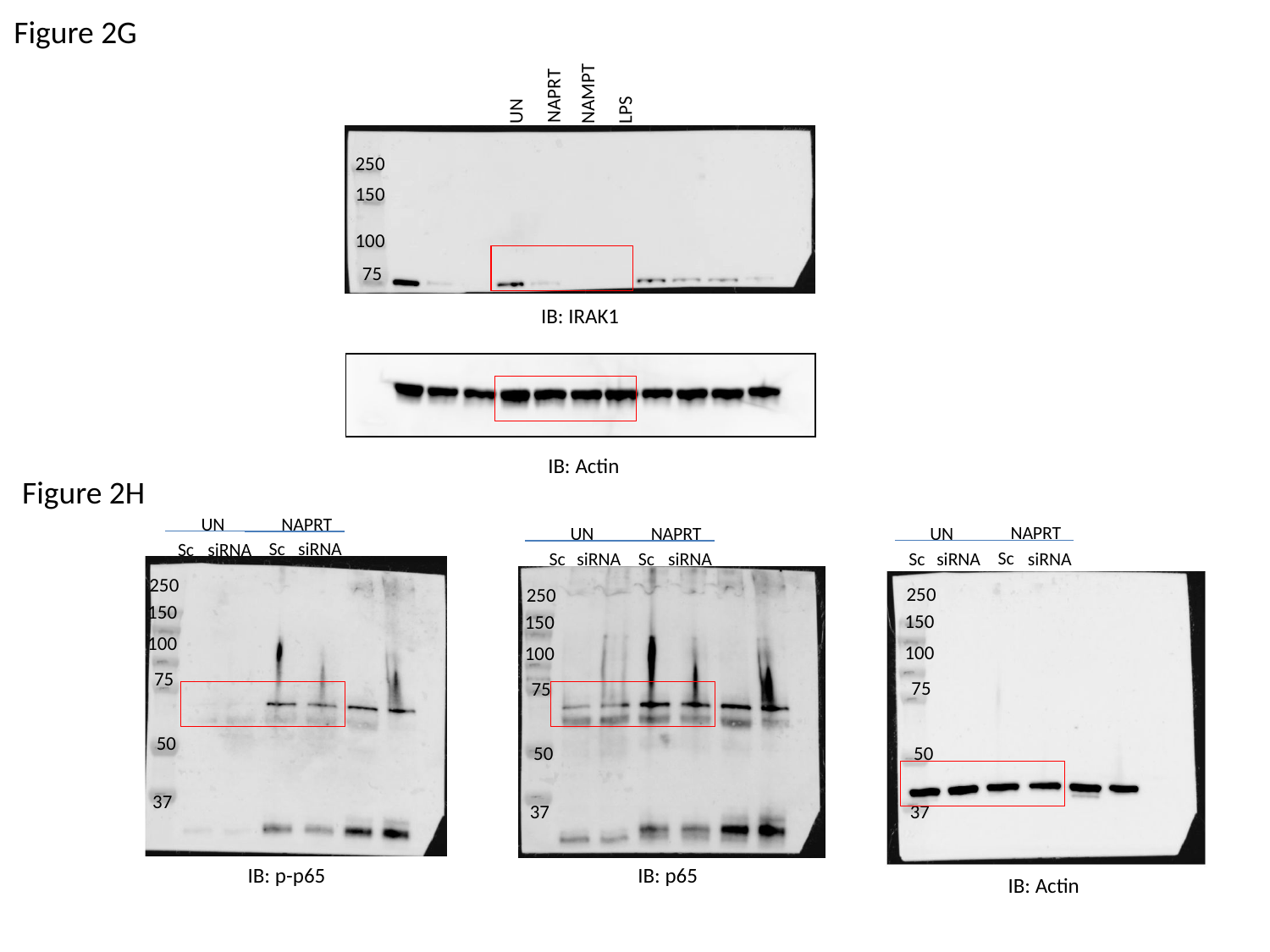

Figure 2G
NAPRT
LPS
UN
NAMPT
250
150
100
75
IB: IRAK1
IB: Actin
Figure 2H
NAPRT
UN
NAPRT
NAPRT
UN
UN
Sc
siRNA
Sc
siRNA
Sc
Sc
Sc
siRNA
siRNA
Sc
siRNA
siRNA
250
250
250
150
150
150
100
100
100
75
75
75
50
50
50
37
37
37
IB: p-p65
IB: p65
IB: Actin

## Slide 5
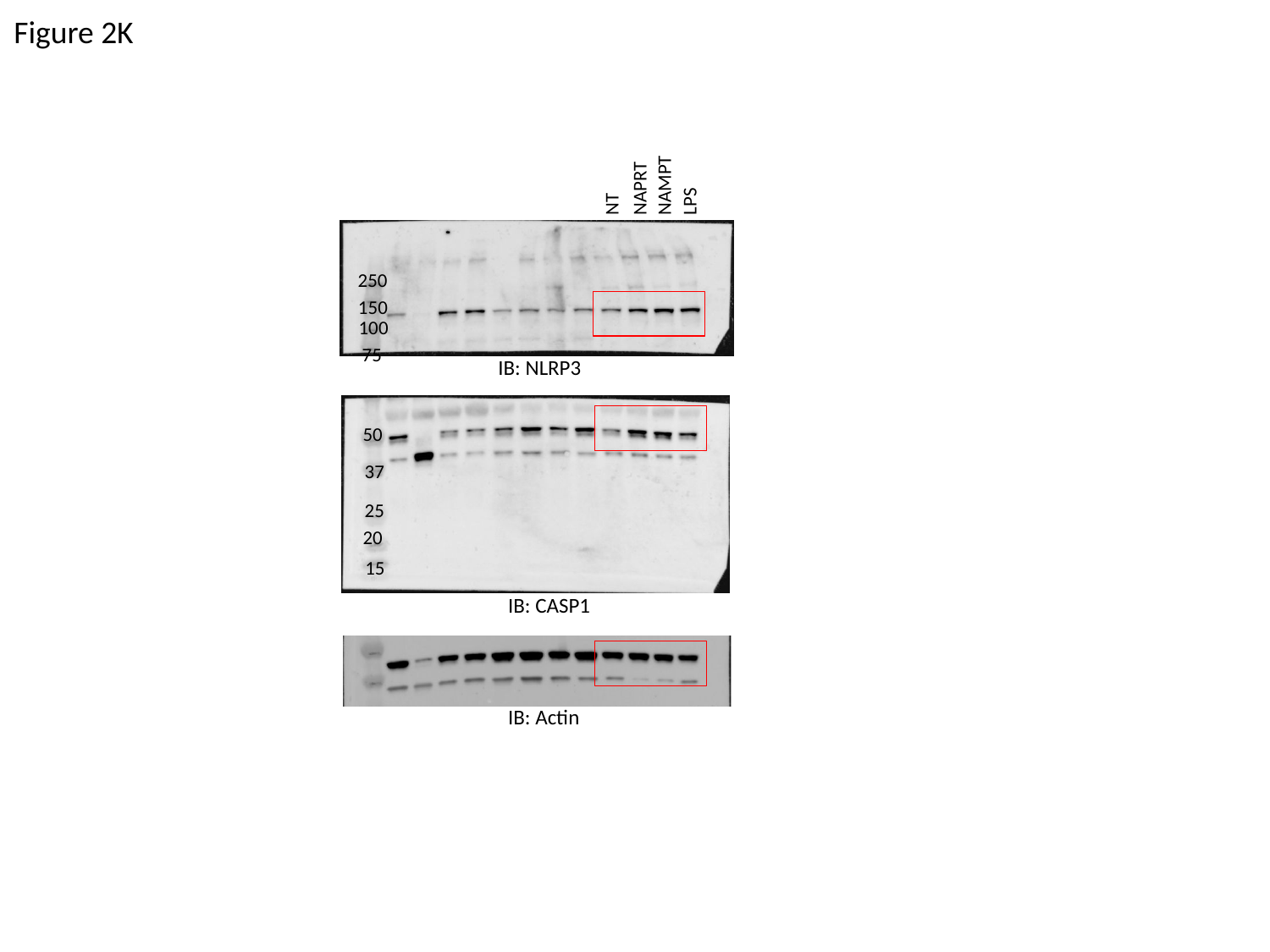

Figure 2K
NAPRT
NAMPT
LPS
NT
250
150
100
75
IB: NLRP3
50
37
25
20
15
IB: CASP1
IB: Actin

## Slide 6
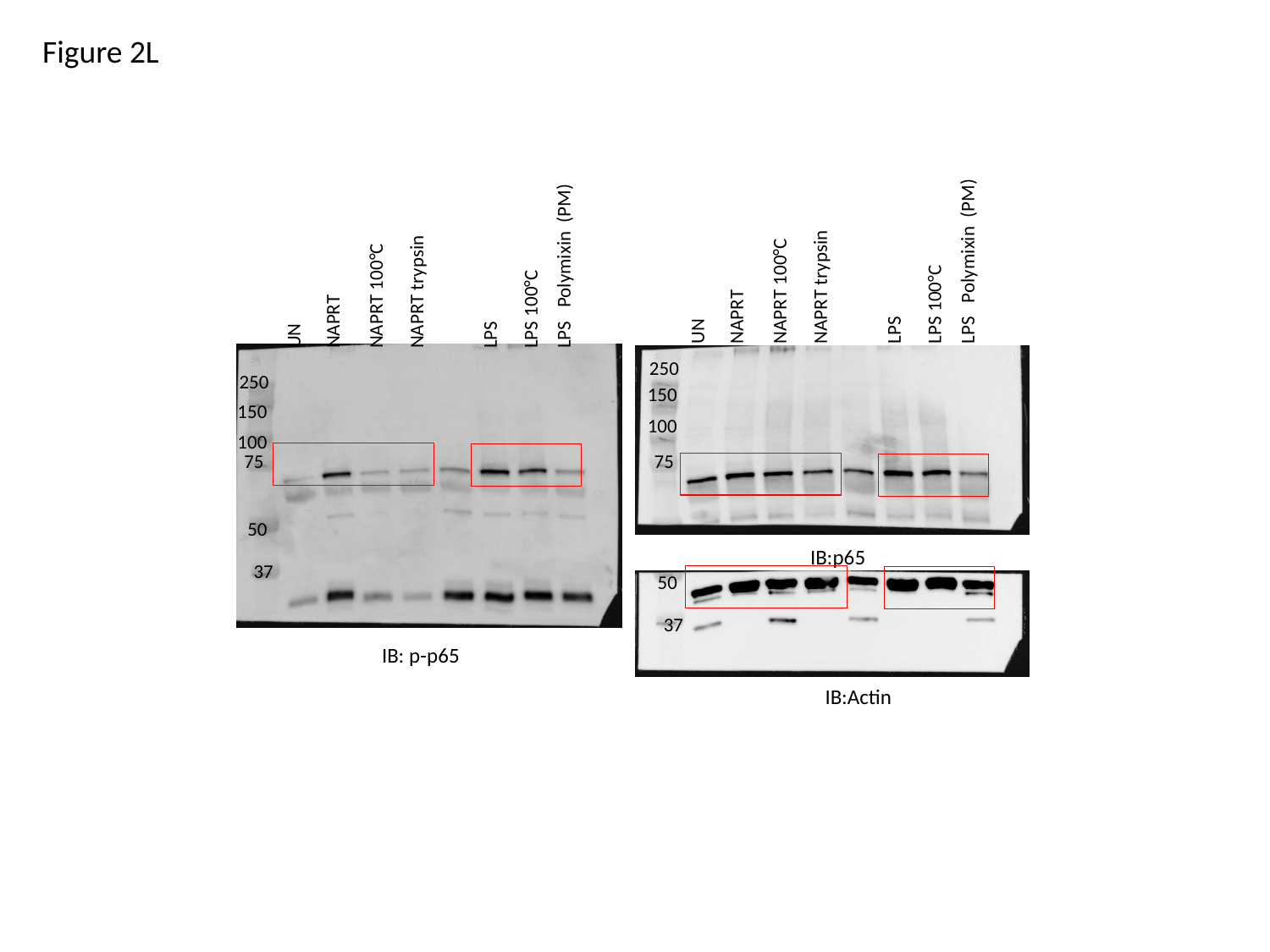

Figure 2L
NAPRT trypsin
LPS Polymixin (PM)
NAPRT trypsin
LPS Polymixin (PM)
NAPRT 100°C
LPS 100°C
NAPRT 100°C
LPS 100°C
NAPRT
LPS
UN
NAPRT
LPS
UN
250
250
150
150
100
100
75
75
50
IB:p65
37
50
37
IB: p-p65
IB:Actin

## Slide 7
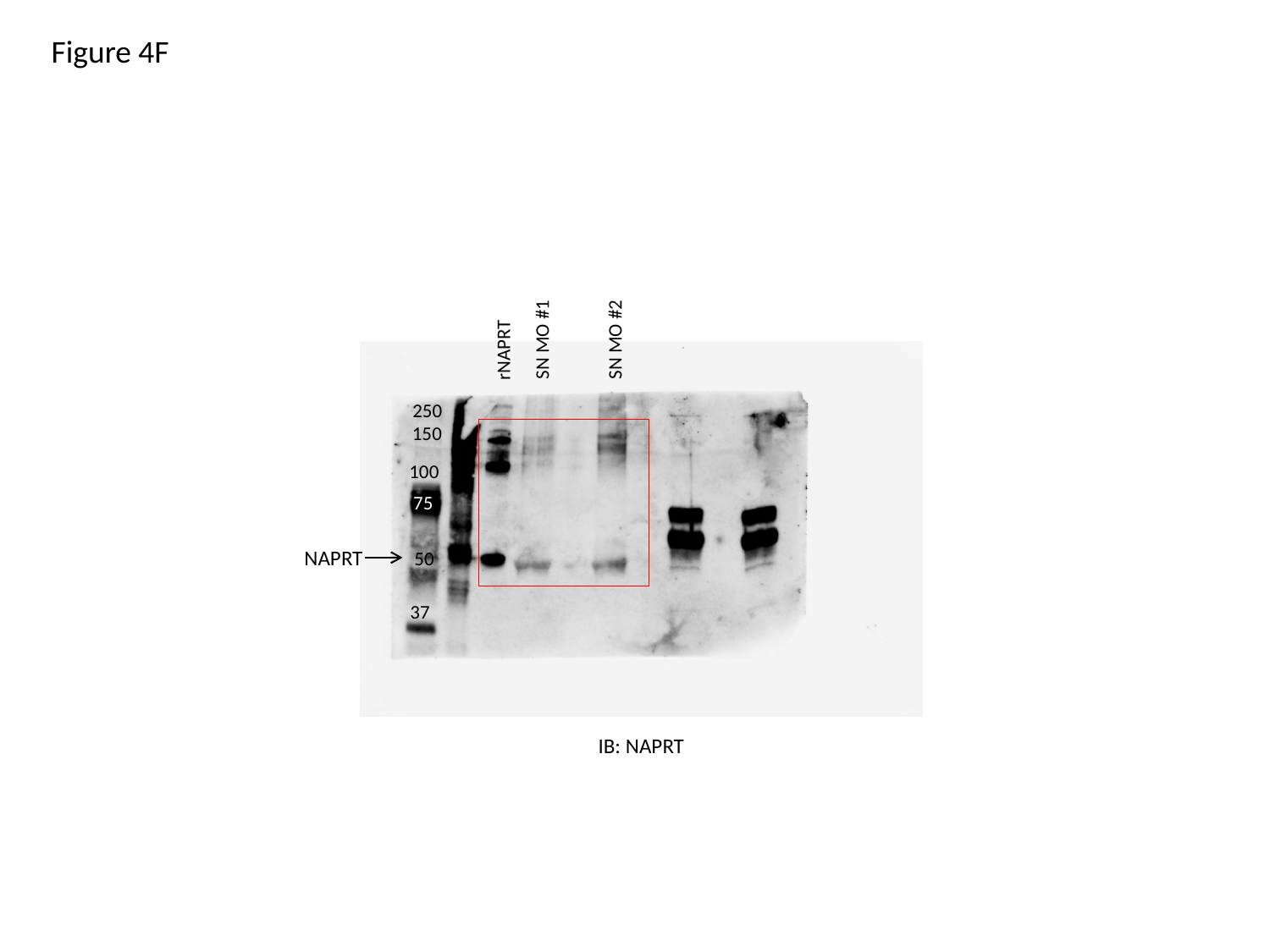

Figure 4F
SN MO #1
SN MO #2
rNAPRT
250
150
100
75
NAPRT
50
37
IB: NAPRT

## Slide 8
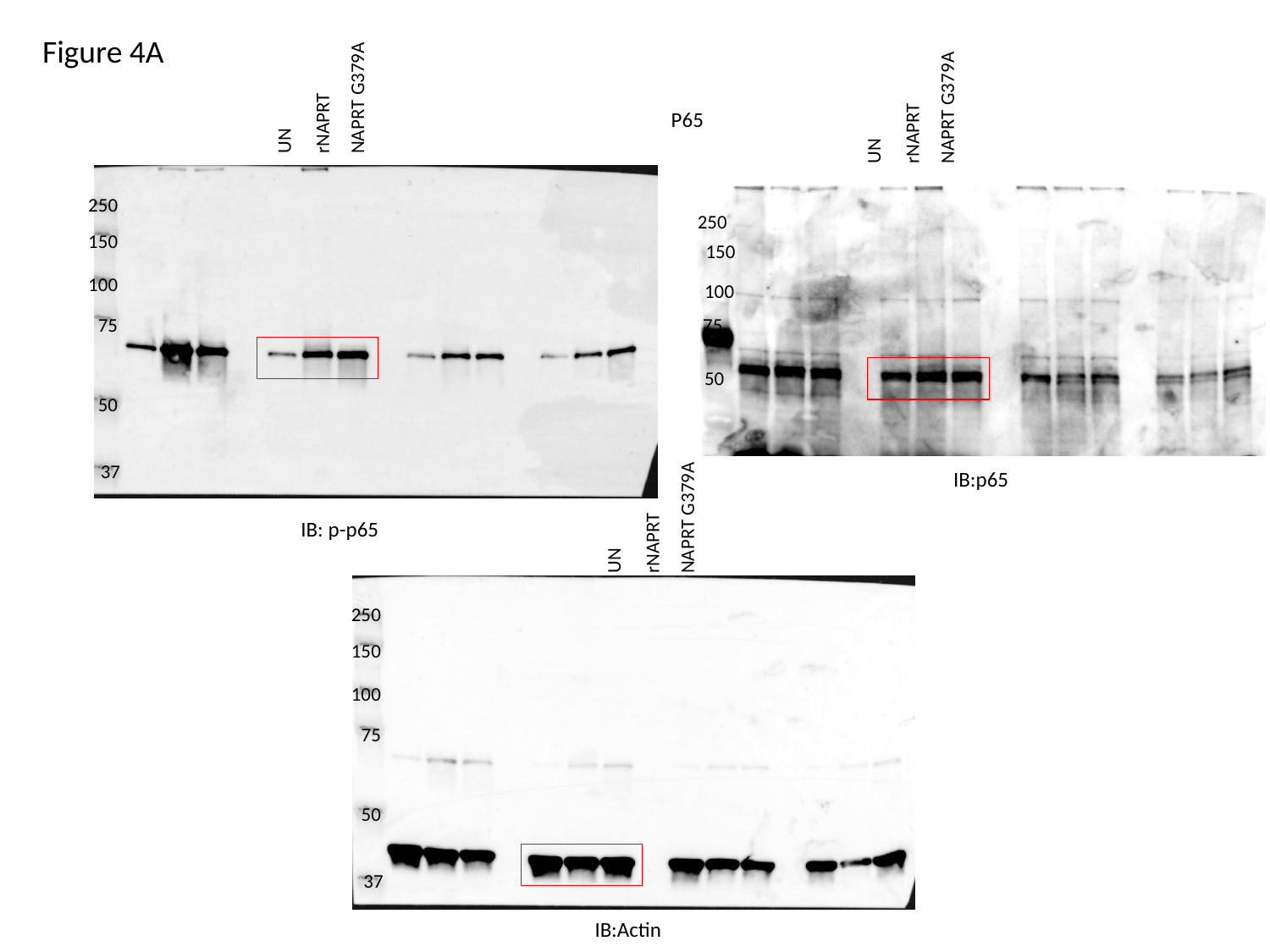

Figure 4A
NAPRT G379A
NAPRT G379A
rNAPRT
UN
P65
rNAPRT
UN
250
250
150
150
100
100
75
75
50
50
37
IB:p65
NAPRT G379A
IB: p-p65
rNAPRT
UN
250
150
100
75
50
37
IB:Actin

## Slide 9
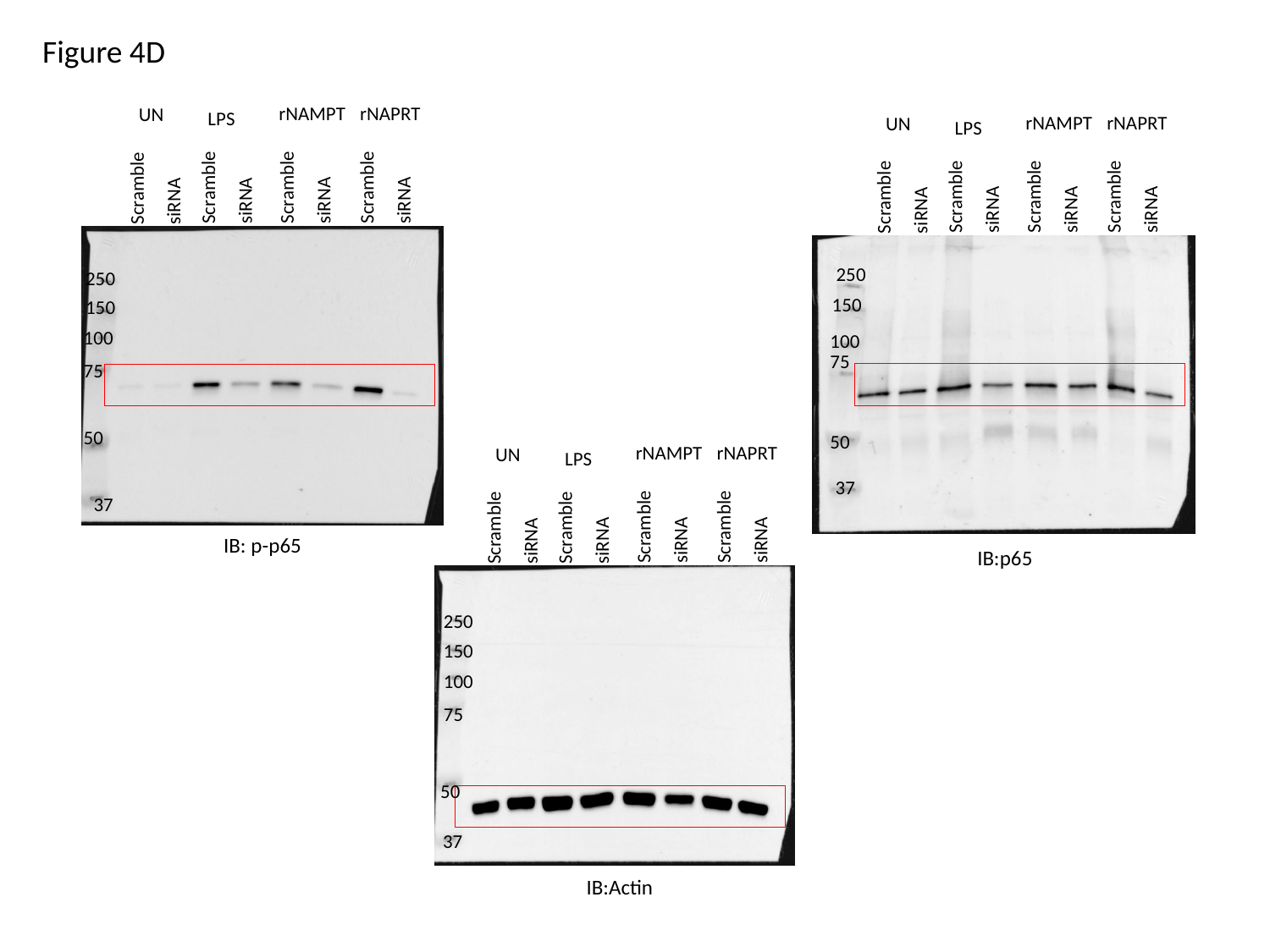

Figure 4D
rNAMPT
rNAPRT
UN
LPS
rNAMPT
rNAPRT
UN
LPS
Scramble
siRNA
Scramble
siRNA
Scramble
siRNA
Scramble
siRNA
Scramble
siRNA
Scramble
siRNA
Scramble
siRNA
Scramble
siRNA
250
250
150
150
100
100
75
75
50
50
rNAMPT
rNAPRT
UN
LPS
Scramble
siRNA
Scramble
siRNA
Scramble
siRNA
Scramble
siRNA
37
37
IB: p-p65
IB:p65
250
150
100
75
50
37
IB:Actin

## Slide 10
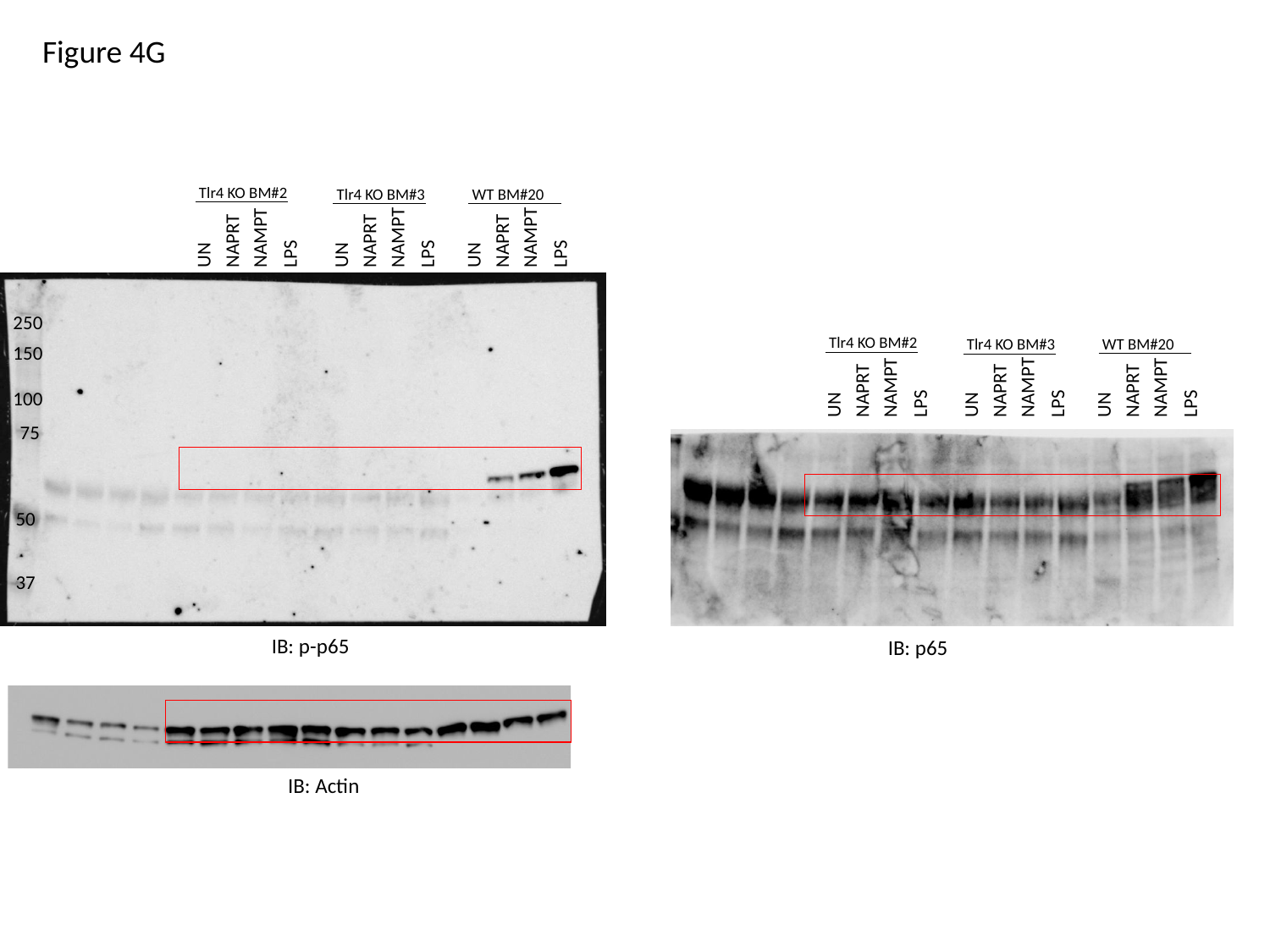

Figure 4G
Tlr4 KO BM#2
WT BM#20
Tlr4 KO BM#3
NAPRT
NAMPT
LPS
NAPRT
NAMPT
NAPRT
NAMPT
UN
LPS
LPS
UN
UN
250
Tlr4 KO BM#2
WT BM#20
Tlr4 KO BM#3
150
NAPRT
NAMPT
LPS
NAPRT
NAMPT
NAPRT
NAMPT
UN
LPS
LPS
UN
UN
100
75
50
37
IB: p-p65
IB: p65
IB: Actin

## Slide 11
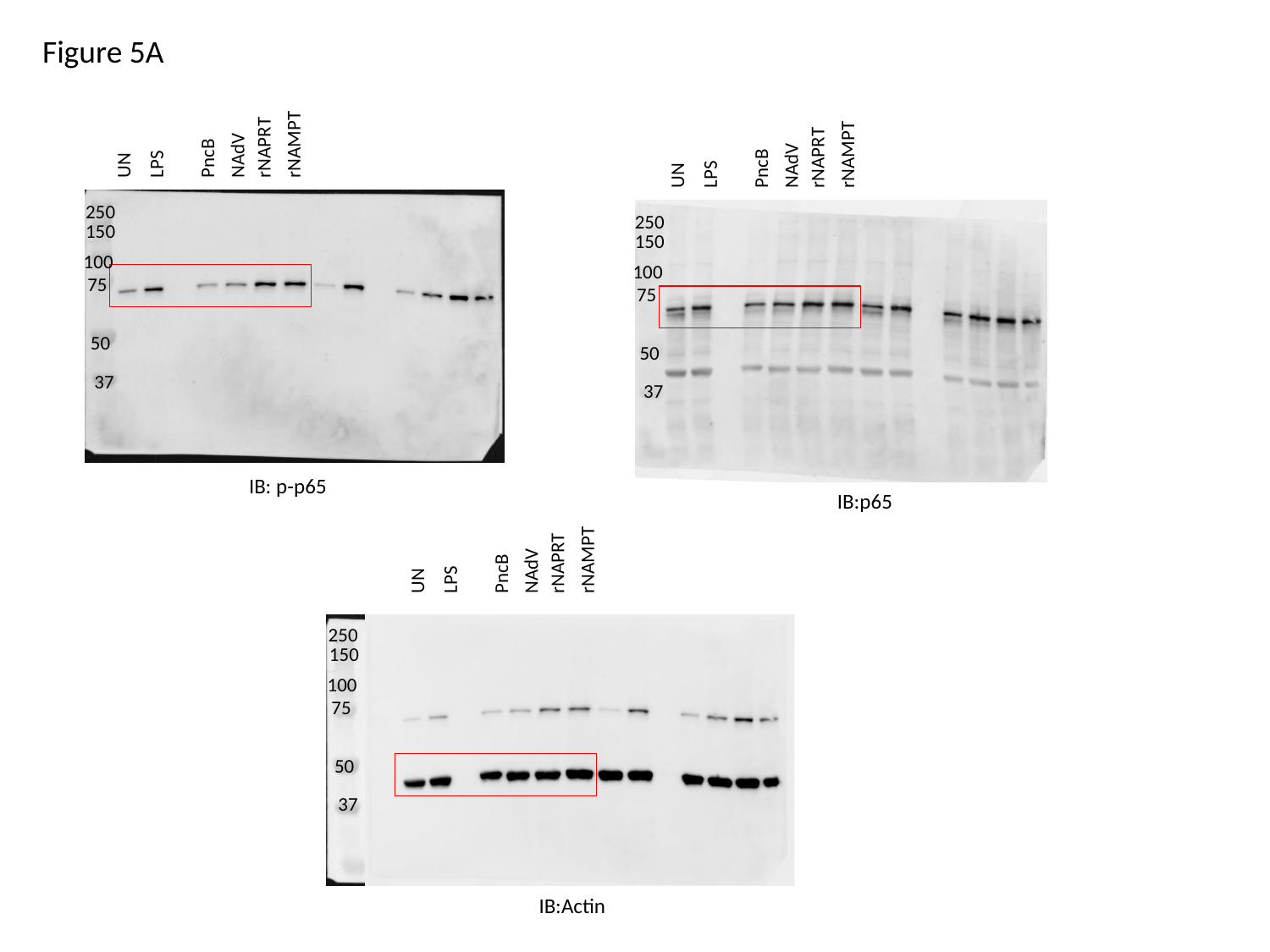

Figure 5A
LPS
PncB
NAdV
rNAPRT
rNAMPT
UN
LPS
PncB
NAdV
rNAPRT
rNAMPT
UN
250
250
150
150
100
100
75
75
50
50
37
37
IB: p-p65
IB:p65
LPS
PncB
NAdV
rNAPRT
rNAMPT
UN
250
150
100
75
50
37
IB:Actin

## Slide 12
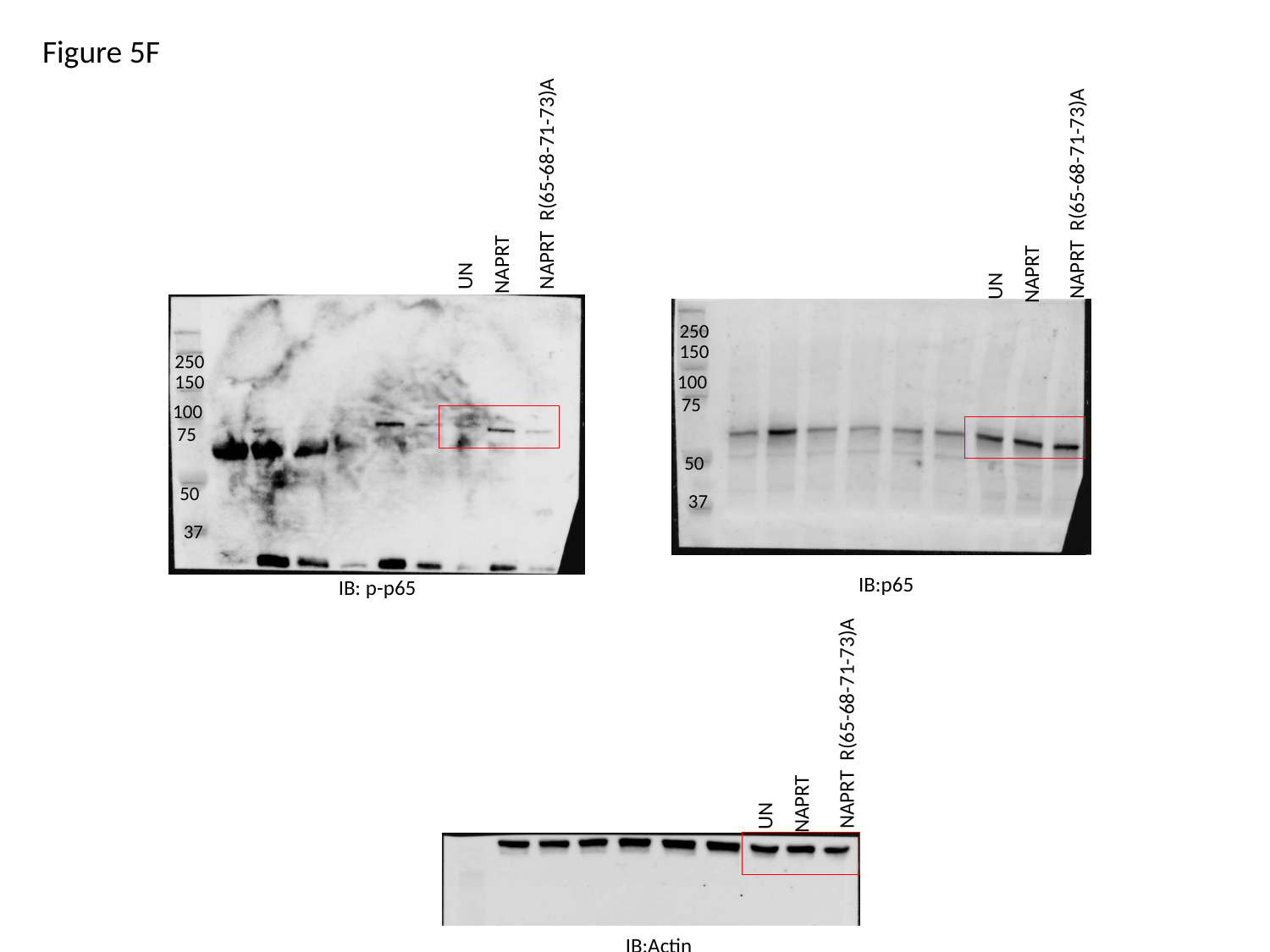

Figure 5F
NAPRT R(65-68-71-73)A
NAPRT R(65-68-71-73)A
NAPRT
NAPRT
UN
UN
250
150
250
150
100
75
100
75
50
50
37
37
IB:p65
IB: p-p65
NAPRT R(65-68-71-73)A
NAPRT
UN
IB:Actin

## Slide 13
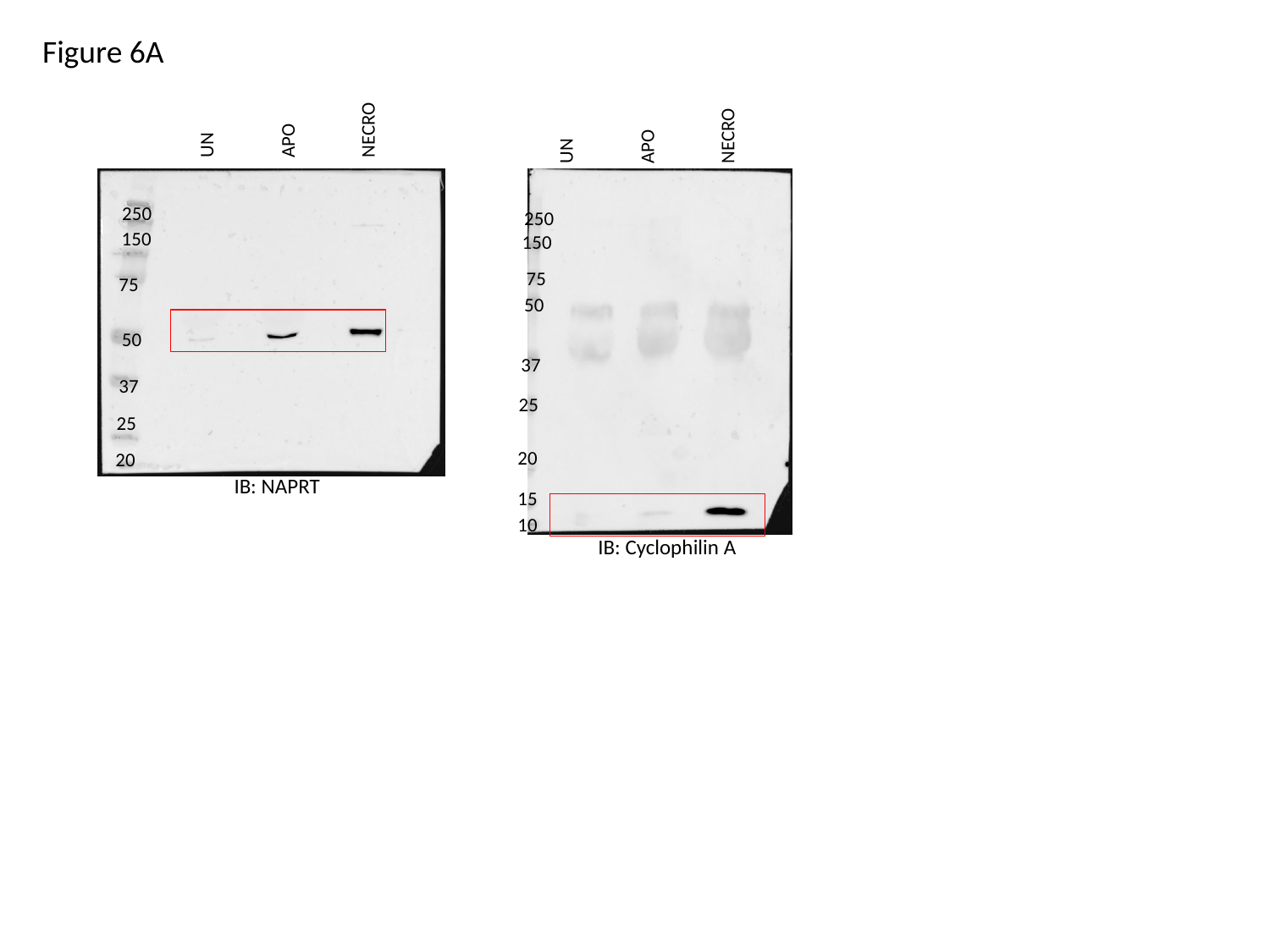

Figure 6A
APO
NECRO
UN
APO
NECRO
UN
250
250
150
150
75
75
50
50
37
37
25
25
20
20
IB: NAPRT
15
10
IB: Cyclophilin A

## Slide 14
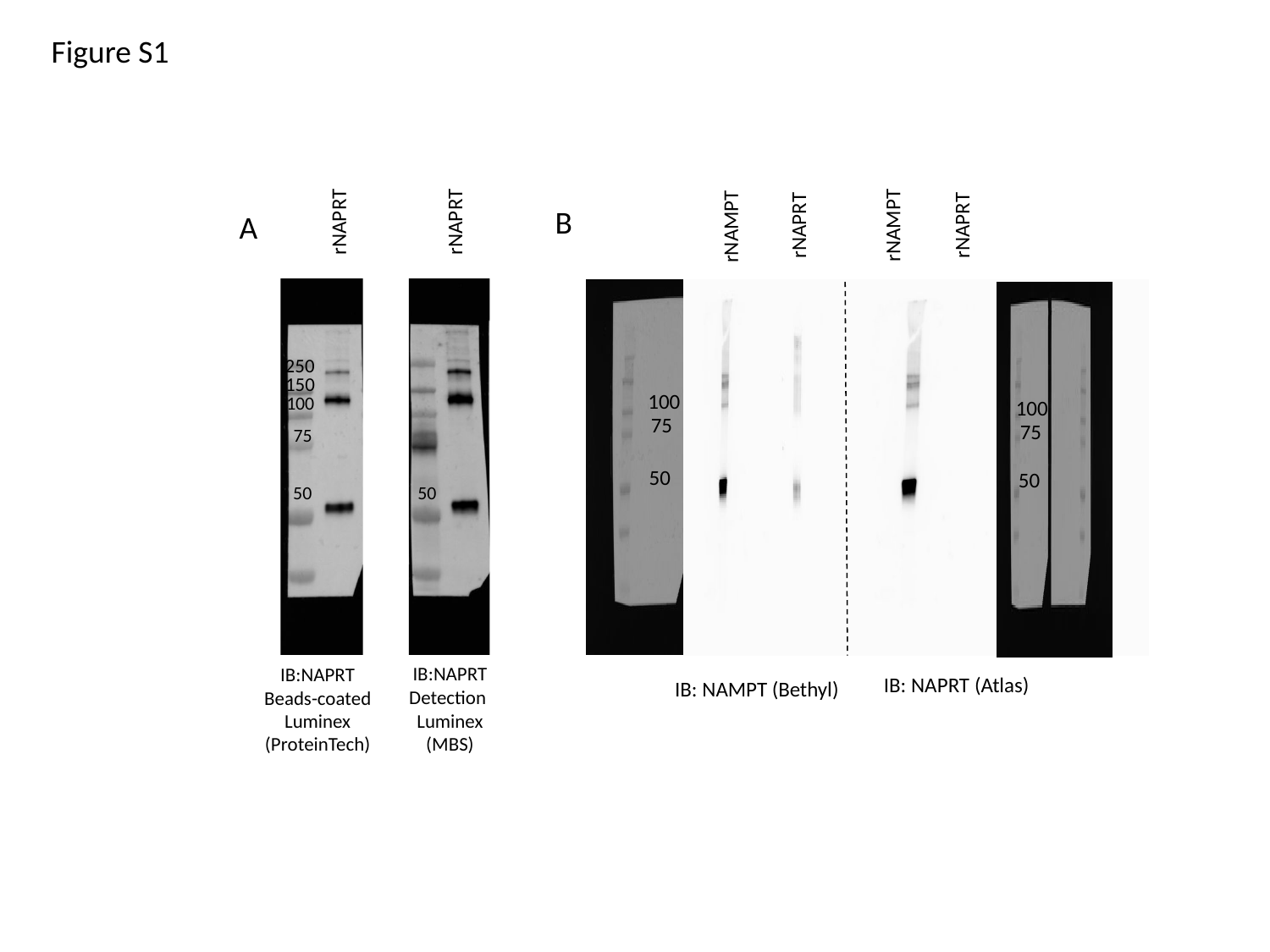

Figure S1
B
A
rNAPRT
rNAPRT
rNAMPT
rNAPRT
rNAPRT
rNAMPT
250
150
100
100
100
75
75
75
50
50
50
50
IB:NAPRT
Detection
Luminex
(MBS)
IB:NAPRT
Beads-coated
Luminex
(ProteinTech)
IB: NAPRT (Atlas)
IB: NAMPT (Bethyl)

## Slide 15
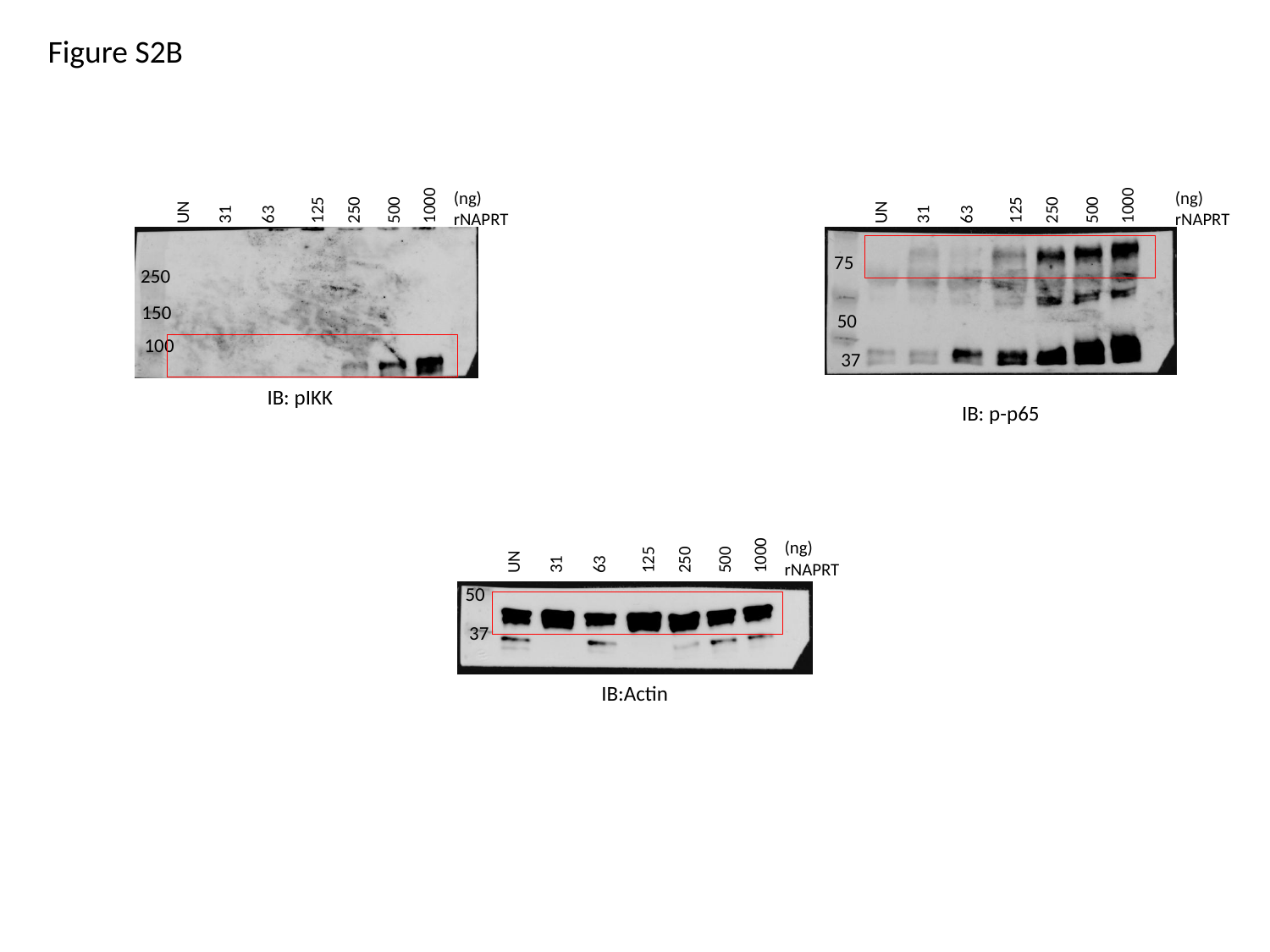

Figure S2B
UN
31
63
125
250
500
1000
UN
31
63
125
250
500
1000
(ng) rNAPRT
(ng) rNAPRT
75
250
150
50
100
37
IB: pIKK
IB: p-p65
UN
31
63
125
250
500
1000
(ng) rNAPRT
50
37
IB:Actin

## Slide 16
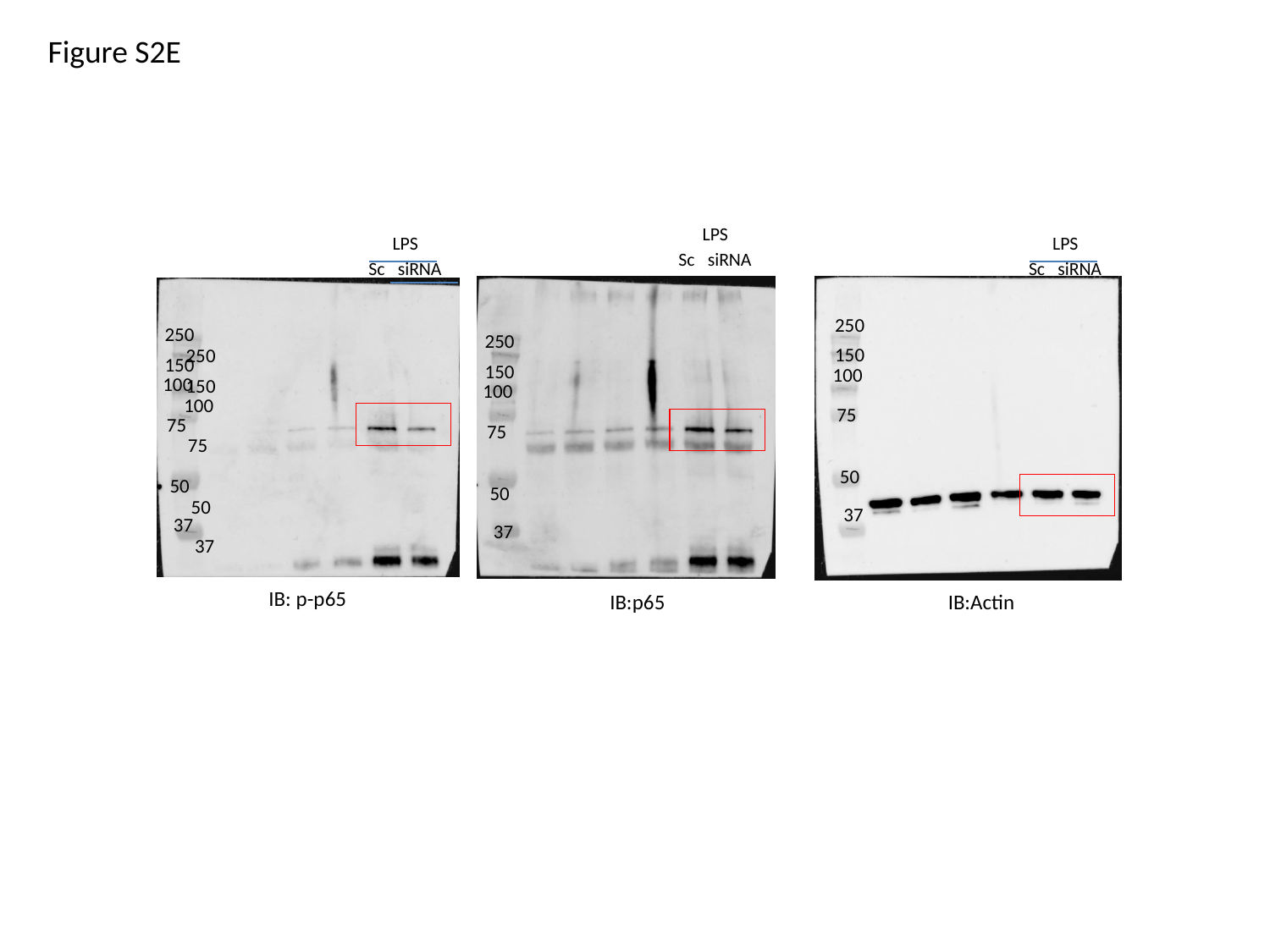

Figure S2E
LPS
LPS
LPS
Sc
siRNA
Sc
siRNA
Sc
siRNA
250
250
250
150
250
150
150
100
100
150
100
100
75
75
75
75
50
50
50
50
37
37
37
37
IB: p-p65
IB:p65
IB:Actin

## Slide 17
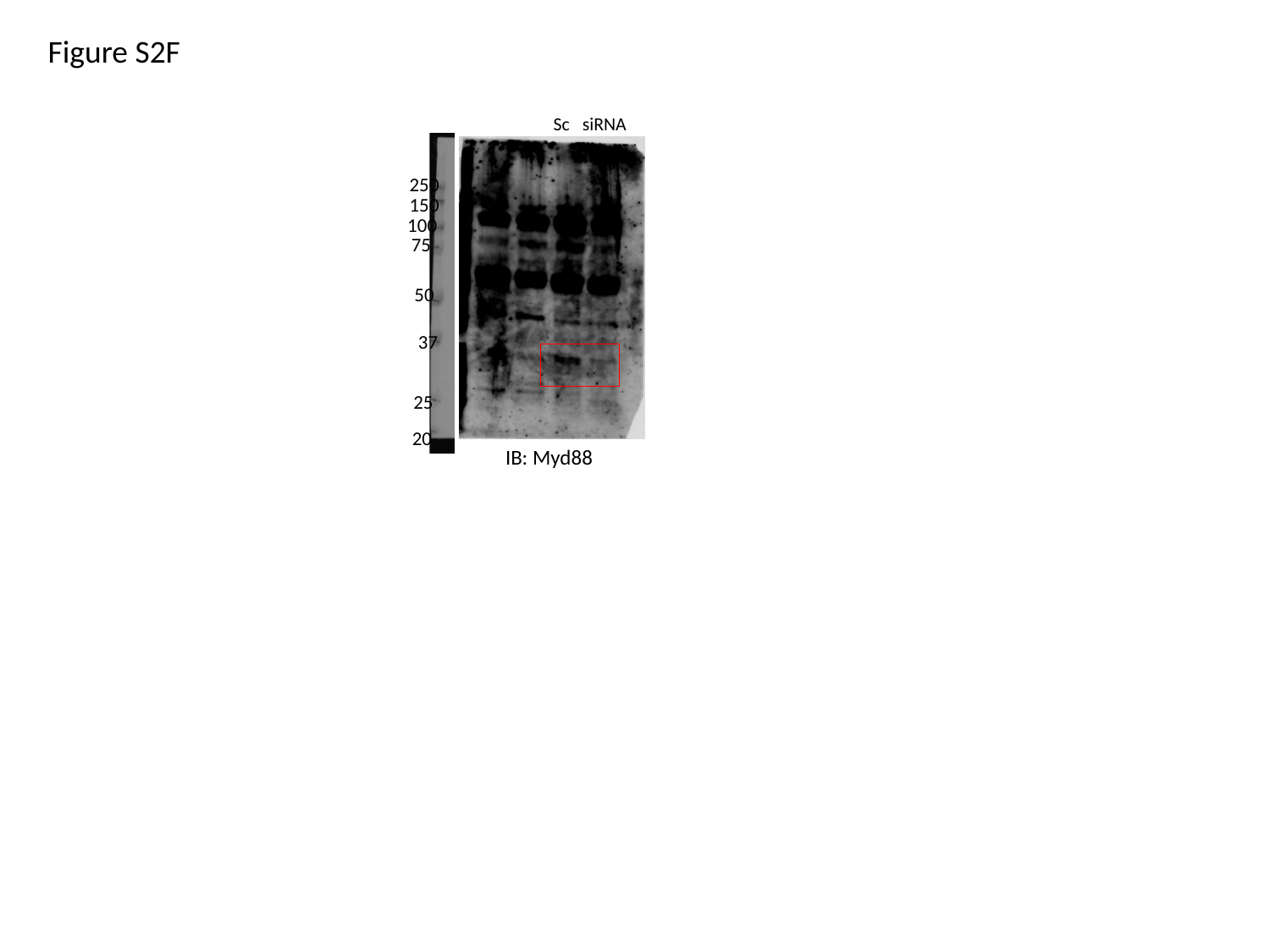

Figure S2F
Sc
siRNA
250
150
100
75
50
37
25
20
IB: Myd88
